# Genetic Variants in *COX2* and *ALOX* Genes and Breast Cancer Risk in White and Black Women

**DOI:** 10.3389/fonc.2021.679998

**Published:** 2021-06-24

**Authors:** Jennifer M. Mongiovi, Chi-Chen Hong, Gary R. Zirpoli, Thaer Khoury, Angela R. Omilian, Bo Qin, Elisa V. Bandera, Song Yao, Christine B. Ambrosone, Zhihong Gong

**Affiliations:** ^1^ Department of Cancer Prevention & Control, Roswell Park Comprehensive Cancer Center, Buffalo, NY, United States; ^2^ Department of Epidemiology and Environmental Health, University at Buffalo, Buffalo, NY, United States; ^3^ Slone Epidemiology Center, Boston University, Boston, NY, United States; ^4^ Department of Pathology, Roswell Park Comprehensive Cancer Center, Buffalo, NY, United States; ^5^ Cancer Epidemiology and Health Outcomes, Rutgers Cancer Institute of New Jersey, New Brunswick, NJ, United States

**Keywords:** breast cancer, Black women, cyclooxygenase 2, arachidonate 12-lipoxygenase, 5-LOX, polymorphism

## Abstract

*COX* and *ALOX* genes are involved in inflammatory processes and that may be related to breast cancer risk differentially between White and Black women. We evaluated distributions of genetic variants involved in *COX2* and *ALOX*-related pathways and examined their associations with breast cancer risk among 1,275 White and 1,299 Black cases and controls who participated in the Women’s Circle of Health Study. Odds ratios (ORs) and 95% confidence intervals (CIs) were estimated using multivariable-adjusted logistic regression models. Our results showed differential associations of certain genetic variants with breast cancer according to menopausal and ER status in either White or Black women. In White women, an increased risk of breast cancer was observed for *COX2*-rs689470 (OR: 2.02, *P* = 0.01) in the dominant model, and was strongest among postmenopausal women (OR: 2.72, *P* = 0.02) and for estrogen receptor positive (ER+) breast cancers (OR: 2.60, *P* = 0.001). A reduced risk was observed for *ALOX5*-rs7099874 (OR: 0.75, *P* = 0.01) in the dominant model, and was stronger among postmenopausal women (OR: 0.68, *P* = 0.03) and for ER+ cancer (OR: 0.66, *P* = 0.001). Four SNPs (rs3840880, rs1126667, rs434473, rs1042357) in the *ALOX12* gene were found in high LD (r^2^ >0.98) in White women and were similarly associated with reduced risk of breast cancer, with a stronger association among postmenopausal women and for ER− cancer. Among Black women, increased risk was observed for *ALOX5*-rs1369214 (OR: 1.44, *P* = 0.003) in the recessive model and was stronger among premenopausal women (OR: 1.57, *P* = 0.03) and for ER+ cancer (OR: 1.53, *P* = 0.003). Our study suggests that genetic variants of *COX2* and *ALOX* genes are associated with breast cancer, and that these associations and genotype distributions differ in subgroups defined by menopausal and ER status between White and Black women. Findings may provide insights into the etiology of breast cancer and areas for further research into reasons for breast cancer differences between races.

## Highlights 

Genetic variants of *COX2* and *ALOX* are associated with breast cancer. These associations and genotype distributions differ in subgroups defined by menopausal and estrogen receptor status between White and Black women.

## Introduction

Among women in the United States, breast cancer is the most common cancer and the second leading cause of cancer death (1). It is well documented that the risk and burden of this disease varies across women of different age, race, ethnicity, and socioeconomic status (2, 3). Although the incidence of breast cancer has historically been higher among White women compared to Black women, incidence rates have converged for the two populations, while the mortality gap is widening (1). Some of this disparity is partly due to differences in access to screening and optimal cancer treatment (4), but Black women are also more likely to be diagnosed with aggressive tumors, i.e., high stage, high grade and negative for estrogen receptor (ER) status (5). Other key factors contributing to these differences are still largely unknown.

One hypothesis is that there may be variation in the molecular and cellular mechanisms in response to chronic inflammation, a process involving the immune system and various inflammatory regulators (6, 7). The common pathological features of chronic inflammation and carcinogenesis include elevation of proinflammatory mediators, such as cytokines, chemokines, prostaglandins and leukotrienes, which orchestrate crosstalk between various cells to create a tumor-supporting microenvironment, and consequently promote tumor initiation, growth and progression (8). Polymorphisms in genes encoding enzymes in these pathways may affect their expression or activity, and ultimately alter an individual’s susceptibility to breast cancer risk. Our studies have identified genetic variants in multiple chemokine- and cytokine-related genes associated with breast cancer risk, with differing associations between White and Black populations (9–11), but prostaglandin- and leukotriene-related pathways remain to be investigated.

Cyclooxygenase 2 (COX-2), also known as prostaglandin-endoperoxide synthase 2 (PTGS2), a key enzyme in prostaglandin synthesis, is known to play a role in carcinogenesis and tumor progression, including breast cancer (12, 13). COX-2 expression is induced by inflammatory stimuli, and aberrant expression is commonly found in epithelial malignancies. Specifically in breast cancer, previous research has shown that overexpression of COX-2 is observed in nearly 60% of invasive breast cancer, while barely detectable in most normal tissues, thus, it may be an early event in mammary tumorigenesis (14–16). Pre-clinical studies have found that COX-2 overexpression can lead to a higher production of prostaglandin E2 (PGE2), an important mediator of inflammation and contributor to immunosuppression, resulting in cell proliferation, apoptosis inhibition, and tumor angiogenesis (16–18). Several variations in *COX*2 gene have been associated with susceptibility to breast cancer (19, 20). In addition to the prostaglandin pathway, the key genes in the arachidonate lipoxygenase (ALOX) pathway, including *ALOX5*, *ALOX5AP*, and *ALOX12*, and associated metabolites, such as leukotrienes, play an important role in inflammation and carcinogenesis (21, 22). However, the impact of both *COX2* and *ALOX* genetic variants have been minimally explored among women of minority ancestral backgrounds and current data are inconclusive.

In this large case-control study, we examined associations between genetic variants of *COX2* and three *ALOX* genes and risk of breast cancer among White and Black women. We further considered if associations varied according to menopausal and ER status. We hypothesized that deviations from the standard distribution of “at-risk” alleles for specific single nucleotide polymorphisms (SNPs) could be associated with risk of breast cancer and may vary between races.

## Materials and Methods

### Study Population

The Women’s Circle of Health Study (WCHS) was a case–control study designed to assess risk factors associated with early onset and aggressive breast cancer among White and Black women. Further details on the study design, enrollment criteria, biospecimen and questionnaire data collection have been previously described (11, 23, 24). Briefly, women with primary incident breast cancer were initially identified using hospital-based case ascertainment in four boroughs of metropolitan New York City (2002–2008) and later through population-based rapid case ascertainment by the New Jersey State Cancer Registry (2006–2012) for 10 counties in New Jersey. Eligible cases were English speaking women 20–75 years of age who self-identified as White or Black and had been recently diagnosed with primary, histologically confirmed breast cancer. Women who had a previous history of cancer other than non-melanoma skin cancer were excluded. Controls were initially recruited from the target population in the same residential area using random digit dialing and then through various community recruitment efforts for Black women (25). Cases and controls were frequency matched based on self-reported race and 5-year age groups. Data and DNA samples from 1,275 White (637 cases, 638 controls) and 1,299 Black (584 cases, 715 controls) participants were included for this analysis. This study was approved by institutional review boards at the Roswell Park Comprehensive Cancer Center, the Rutgers Cancer Institute of New Jersey, Mount Sinai School of Medicine (now the Icahn School of Medicine at Mount Sinai), and participating hospitals in New York City. Signed informed consent was obtained from each participant prior to interview and biospecimen collection.

### Data and Sample Collection

Detailed data on demographic characteristics, medical history, family history of cancer, lifestyle factors, and anthropometric measures were collected in-person by trained interviewers. Blood samples, as a source of DNA, were initially collected from approximately 850 participants until we transitioned to collection of saliva samples using Oragene™ kits (DNA Genotek Inc., Kanaya, Ontario, Canada) as a source of DNA. Pathology data including ER status, grade, and stage were collected and abstracted from patient records by trained staff.

Genomic DNA was extracted from blood samples using FlexiGene™ DNA isolation kits (Qiagen Inc., Valencia, CA) and from saliva samples using Oragene™ kits. DNA was then evaluated and quantified by Nanodrop UV-spectrometer (Thermo Fisher Scientific Inc., Wilmington, DE) and PicoGreen-based fluorometric assay (Molecular Probes, Invitrogen Inc., Carlsbad, CA). Samples were stored at −80°C until analysis.

### SNP Selection and Genotyping

We surveyed the Human Genome Epidemiology (HuGE) Navigator for the four genes involved in *COX-2* and *ALOX* inflammation-related pathways to identify SNPs that were previously associated with risk of any cancer or cancer outcome, with a focus on SNPs that were previously shown to be functional (26). A panel of 31 SNPs were then selected and genotyped at the Genomics Shared Resource at Roswell Park using the Illumina GoldenGate assay (Illumina Inc., San Diego, CA). To account for population admixture in the analysis, all samples were also genotyped for a panel of 100 ancestry informative markers (AIMs) that were previously validated in the Black Women’s Health Study (27). Proportions of European Ancestry and African Ancestry of individual White and Black women were computed quantitatively using the Bayesian Markov Chain Monte Carlo clustering algorithm implemented in STRUCTURE, based on data from the 100 genotyped AIMs. Since the sum of two ancestral proportions in each individual is always one, we used only the proportion of European Ancestry in all analyses (28). As a quality control measure in both genotyping efforts, 5% duplicates and two sets of in-house trio samples were included across all plates. One SNP (*COX2-*rs20417) was removed due to the violation of Hardy–Weinberg Equilibrium, all other SNPs were included in the analysis.

### Statistical Analysis

Descriptive analysis was done using chi-square tests for categorical variables and t-tests for continuous variables between 1,275 White and 1,299 Black cases and controls. Odds ratios (ORs) and 95% confidence intervals (CIs) for each SNP were derived from multivariable logistic regression models with adjustment for age (continuous), proportion of European ancestry (continuous), and family history of breast cancer (yes, no). Other covariates did not significantly affect the risk estimates and thus were not included in the multivariable-adjusted analysis. Participants with the most common homozygous genotype among White controls were treated as the referent group. Genotypic (co-dominant) models were assumed for SNP effects. Based on the risk estimates, heterozygotes were combined with either homozygous rare or homozygous common genotypes to explore dominant and recessive models, respectively. Additive genotype coding on the number of rare alleles was analyzed as an ordinal variable in tests for linear trend. Analysis was performed separately for White and Black women, and interactions by race were tested by including an interaction term SNP*race in multivariable logistic models and performing the likelihood ratio test. Additional stratified analyses were conducted to examine whether SNP associations with breast cancer risk differed by menopausal or ER status.

All analyses were conducted using SAS V 9.4 (SAS Institute, Cary, NC). Linkage disequilibrium (LD) was determined by calculating r^2^ values between SNP pairs using Haploview (29). Statistical tests were two-sided and considered statistically significant for uncorrected *P <*0.05. All significant p-values were further adjusted for multiple comparisons using Bonferroni correction, with *P <*0.002 (0.05/30) considered statistically significant.

## Results

### Participant Characteristics

Characteristics of White and Black cases and controls are shown in **Table 1**. In both races, cases were slightly older and were more likely to have a history of benign breast disease. Compared with White controls, cases had lower proportion of European ancestry, received less college and post-graduate education, and were more likely to have a family history of breast cancer. Among Black women, cases were more likely to be postmenopausal. As expected, Black cases were more likely than White cases to be diagnosed with ER negative breast cancer (32.1% *vs.* 18.8%).

**Table 1:** Characteristics of 1,275 White and 1,299 Black cases and controls in the WCHS^a^.

Characteristics	White			Black		
	Cases (n = 637)	Controls (n = 638)	*P-value* ^c^	Cases (n = 584)	Controls (n = 715)	*P-value* ^c^
Age (yr), mean (SD)^b^	52.2 (10.0)	49.7 (8.7)	**<0.0001**	51.7 (10.4)	48.7 (9.5)	**<0.0001**
Body mass index, kg/m^2^, mean (SD)^b^	27.3 (6.6)	27.4 (7.1)	0.76	31.2 (6.8)	31.9 (7.9)	0.06
% European ancestry^b^	96.7 (8.1)	98.5 (3.7)	**<0.0001**	14.1 (16.1)	13.9 (13.9)	0.89
Menopausal status, n (%)			0.30			**0.03**
Premenopausal	331 (52.0)	350 (54.9)		286 (49.0)	393 (55.0)	
Postmenopausal	306 (48.0)	288 (45.1)		298 (51.0)	322 (45.0)	
Family history of breast cancer in first degree relative, n (%)			**0.0006**			0.13
No	481 (75.5)	533 (83.5)		498 (85.3)	630 (88.1)	
Yes	156 (24.5)	105 (16.5)		86 (14.7)	85 (11.9)	
Education, n (%)			**<0.0001**			0.42
≤high school	19 (3.0)	6 (0.9)		80 (13.7)	95 (13.3)	
High school graduate or equivalent	112 (17.6)	65 (10.2)		178 (30.5)	187 (26.2)	
Some college	140 (22.0)	113 (17.7)		159 (27.2)	201 (28.1)	
College graduate	198 (31.0)	208 (32.6)		102 (17.5)	139 (19.4)	
Post-graduate degree	168 (26.4)	246 (38.6)		65 (11.1)	93 (13.0)	
History of benign breast disease, n (%)			**0.0006**			**<0.0001**
No	368 (58.4)	431 (67.8)		399 (68.6)	564 (79.0)	
Yes	262 (41.6)	205 (32.2)		183 (31.4)	150 (21.0)	
Estrogen receptor (ER) Status, n (%)^d^			–			–
Positive	450 (81.2)			351 (67.9)		
Negative	104 (18.8)			166 (32.1)		

a Number may not add up to the total number due to missing values.

b SD, standard deviation.

c P-value were from t-test for continuous variables and Chi-square test for categorical variables.

d ER status were available for 554 (87.0%) White cases and 517 (88.5%) Black cases. Bold, significant P-values.

### Associations of SNPs With Breast Cancer Risk in White and Black Women

Genotype distributions of each SNP and their associations with overall breast cancer risk in White and Black women are shown in **Supplemental Table S1**. We observed differences in genotype distributions between White and Black populations, and for seven of these SNPs, the minor allele variant was reversed between the two groups. A number of SNPs were statistically significantly associated with overall breast cancer risk in either AA or EA women, with results shown in **Table 2**. In White women, an increased risk of breast cancer was observed for *COX2*-rs689470 (OR: 2.02, 95% CI: 1.16–3.53) in the dominant model and *ALOX5*-rs1487562 (OR: 1.80, 95% CI: 1.02–3.18) in the recessive model, while the association among Black women was essentially null. A reduced risk among White women was also observed for *ALOX5*-rs7099874 (OR: 0.75, 95% CI: 0.60–0.94) in the dominant model. Four SNPs (rs3840880, rs1126667, rs434473, rs1042357) in the *ALOX12* gene were in high LD (r^2^ >0.98) and were similarly associated with reduced risk in White women. These SNPs, however, were not in LD (r^2^ <0.43) in the Black population and were not associated with overall breast cancer risk. Among Black women, significant associations were observed for *ALOX5*-rs1369214 (OR: 1.44, 95% CI: 1.13–1.84) and rs1051713 (OR: 0.53, 95% CI: 0.28–0.98) in the recessive models. Although these genotype-breast cancer associations differed in strength according to race, significant SNP-race interactions were observed only for *COX2*-rs689470 and *ALOX5*-rs1487562 (*P*-interaction = 0.006 and 0.03, respectively), as shown above, both SNPs were associated with increased risk of breast cancer among White women.

**Table 2:** SNPs of inflammation-related pathways and risk of breast cancer among White and Black women in the WCHS.

Gene	SNP	Chr	Coordinate	Genotype	White			Black			
					# Case/Control	OR (95% CI)^a, b^	*P* ^c, d, e^	# Case/Control	OR (95% CI)^a, b^	*P* ^c,d,e^	*P* ^f^
*COX2*	rs689470	1	186641058	CC	589/618	1.00 (ref)	**0.005**	205/262	1.00 (ref)	0.86	**0.006**
				CT	47/17	2.37 (1.32–4.27)		273/325	1.07 (0.83–1.38)		
				TT	1/3	0.05 (0.00–2.19)		102/127	1.06 (0.77–1.48)		
				CT/TT *vs.* CC	48/20	2.02 (1.16–3.53)	**0.01**	375/452	1.07 (0.84–1.35)	0.58	
*ALOX5*	rs1369214	10	45900729	GG	195/190	1.00 (ref)	0.83	112/156	1.00 (ref)	**0.01**	0.08
				GA	316/310	1.03 (0.79–1.33)		274/367	1.04 (0.78–1.40)		
				AA	123/134	0.94 (0.68–1.30)		196/189	1.48 (1.08–2.04)		
				AA *vs.* GG/GA	511/500	0.92 (0.69–1.12)	0.57	386/523	1.44 (1.13–1.84)	**0.003**	
*ALOX5*	rs1487562	10	45928822	CC	419/428	1.00 (ref)	0.13	337/400	1.00 (ref)	0.33	**0.03**
				CT	180/190	1.01 (0.78–1.29)		211/258	0.97 (0.77–1.23)		
				TT	37/20	1.80 (1.02–3.20)		34/57	0.71 (0.45–1.12)		
				TT *vs.* CC/CT	599/618	1.80 (1.02–3.18)	**0.04**	548/658	0.72 (0.46–1.12)	0.14	
*ALOX5*	rs7099874	10	45928911	GG	328/281	1.00 (ref)	**0.02**	429/525	1.00 (ref)	0.99	0.15
				GC	249/304	0.71 (0.56–0.9)		133/165	0.98 (0.75–1.28)		
				CC	55/48	0.98 (0.64–1.51)		17/19	1.00 (0.51–1.98)		
				GC/CC *vs.* GG	304/352	0.75 (0.60–0.94)	**0.01**	150/184	0.98 (0.76–1.27)	0.89	
*ALOX5*	rs1051713	10	45938746	CC	437/440	1.00 (ref)	0.41	393/486	1.00 (ref)	0.10	0.07
				CT	173/180	0.98 (0.76–1.26)		174/193	1.10 (0.85–1.40)		
				TT	24/16	1.54 (0.80–2.98)		15/34	0.54 (0.29–1.02)		
				TT *vs.* CC/CT	610/620	1.55 (0.81–2.99)	0.19	567/679	0.53 (0.28–0.98)	**0.04**	
*ALOX12*	rs3840880^g^	17	6897844	TT	221/187	1.00 (ref)	0.08	133/159	1.00 (ref)	0.94	0.19
				TG	313/316	0.88 (0.68–1.13)		279/345	0.96 (0.73–1.28)		
				GG	101/131	0.68 (0.49–0.95)		171/211	1.01 (0.74–1.38)		
				GG *vs.* TT/TG	534/503	0.74 (0.55–0.99)	**0.04**	412/504	1.03 (0.74–1.45)	0.80	

a OR, odds ratio; 95%CI, 95% confidence interval.

b Adjusted for age, family history of breast cancer in a first-degree relative, and proportion of European ancestry.

c P-trend for genetic dose response determined by coding genotypes as having 0, 1, or 2 variant allele, which was subsequently analyzed as an ordinal variable.

d P for heterogeneity from dominant or recessive models.

e All significant p-values were further adjusted for multiple comparisons using Bonferroni correction, with P <0.002 (0.05/30) considered statistically significant.

f P for interaction term including genotype and race in the multivariable logistic model.

g Several SNPs on the ALOX12 gene, rs3840880, rs1126667, rs434473, rs1042357, were found in high LD in White women (r^2^ >0.98), but not in LD in Black women (r^2^ <0.43). Bold, significant P-values.

In stratified analyses, associations between each SNP and breast cancer risk were examined separately in pre- and post-menopausal women (**Supplemental Table S2**). In these analyses, a number of SNPs were significantly associated with breast cancer risk in pre- or post-menopausal women in either White or Black women, with results shown in **Table 3**. Among pre-menopausal White women, *ALOX5AP*-rs9315048 was associated with increased risk of breast cancer (OR: 2.11, 95% CI: 1.05–4.28) in the recessive model. Among postmenopausal White women, *COX2*-rs689470 and *ALOX12*-rs2292350 were associated with increased risk (OR: 2.72, 95% CI: 1.16–6.40 and OR: 1.50, 95% CI: 1.04–2.16, respectively) in the dominant models, while *ALOX5*-rs7099874 was associated with reduced risk (OR: 0.68, 95% CI: 0.48–0.96) in the dominant model. Further, for the four *ALOX12-*SNPs in LD that showed similar significant associations with overall breast cancer risk among White women, the reduced risk was stronger among post-menopausal women, as shown for rs3840880 (OR: 0.57, 95% CI: 0.40–0.83). Among pre-menopausal Black women, an increased risk was observed for *ALOX5*-rs1369214 (OR: 1.57, 95% CI: 1.05–2.37) and *ALOX5AP*-rs9315045 (OR: 1.39, 95% CI: 1.01–1.93) in the dominant models, whereas a reduced risk was observed for *ALOX5*-rs1051713 (OR: 0.35, 95% CI: 0.13–0.95) in the recessive model. In post-menopausal Black women, a reduced risk was observed for *COX2*-rs2745557 (OR: 0.63, 95% CI: 0.44-0.92) and *ALOX5AP-*rs4293222 (OR: 0.45, 95% CI: 0.23–0.92) in the dominant models.

**Table 3:** SNPs of inflammation related pathways and risk of breast cancer by menopausal status in the WCHS.

Gene	SNP	Genotype	White						Black					
			Pre-menopausal women			Post-menopausal women			Pre-menopausal women			Post-menopausal women		
			# Case/Control	OR (95% CI)^a, b^	*P* ^c, d, e^	# Case/Control	OR (95% CI) ^a, b^	*P* ^c, d, e^	# Case/Control	OR (95% CI) ^a, b^	*P* ^c, d, e^	# Case/Control	OR (95% CI) ^a, b^	*P* ^c, d, e^
*COX2*	rs689470	CC	310/338	1.00 (ref)	0.07	279/280	1.00 (ref)	**0.02**	103/158	1.00 (ref)	0.49	102/104	1.00 (ref)	0.99
		CT	20/9	2.01 (0.86-4.69)		27/8	2.72 (1.16–6.40)		124/160	1.23 (0.87–1.74)		149/165	1.00 (0.69–1.44)	
		TT	1/3	0.03 (0.00–2.24)		0/0			56/74	1.19 (0.77–1.84)		46/53	0.96 (0.58–1.60)	
		CT/TT *vs.* CC	21/12	1.46 (0.67–3.19)	0.34	27/8	2.72 (1.16–6.40)	**0.02**	180/234	1.22 (0.88–1.68)	0.23	195/218	0.99 (0.69–1.41)	0.95
*COX2*	rs2745557	GG	214/223	1.00 (ref)	0.27	190/174	1.00 (ref)	0.84	200/284	1.00 (ref)	0.69	230/219	1.00 (ref)	**0.03**
		GA	97/101	1.07 (0.76–1.52)		100/102	0.94 (0.65–1.35)		76/94	1.14 (0.80–1.63)		61/96	0.60 (0.41–0.88)	
		AA	18/26	0.61 (0.31–1.18)		16/11	1.20 (0.52–2.78)		9/15	0.85 (0.36–1.99)		7/6	1.16 (0.38–3.58)	
		GA/AA *vs.* GG	115/127	0.97 (0.70–1.34)	0.85	116/113	0.97 (0.68–1.37)	0.85	85/109	1.10 (0.79–1.55)	0.58	68/102	0.63 (0.44–0.92)	**0.02**
*ALOX5*	rs1369214	GG	100/101	1.00 (ref)	0.94	95/89	1.00 (ref)	0.61	42/87	1.00 (ref)	**0.02**	70/69	1.00 (ref)	0.18
		GA	158/172	0.96 (0.67–1.38)		158/138	1.18 (0.80–1.75)		140/197	1.39 (0.90–2.14)		134/170	0.81 (0.53–1.22)	
		AA	72/76	0.92 (0.59–1.43)		51/58	0.99 (0.59–1.63)		102/108	1.90 (1.20–3.01)		94/81	1.15 (0.72–1.82)	
		GA/AA *vs.* GG	230/248	0.95 (0.67–1.33)	0.75	209/196	1.13 (0.78–1.63)	0.53	242/305	1.57 (1.05–2.37)	**0.03**	228/251	0.92 (0.62–1.36)	0.67
*ALOX5*	rs7099874	GG	167/161	1.00 (ref)	0.52	161/120	1.00 (ref)	**0.006**	219/293	1.00 (ref)	0.76	210/232	1.00 (ref)	0.72
		GC	139/157	0.87 (0.63–1.21)		110/147	0.60 (0.42–0.86)		58/88	0.87 (0.60–1.27)		75/77	1.17 (0.80–1.72)	
		CC	22/30	0.74 (0.40–1.36)		33/18	1.29 (0.67–2.47)		6/9	0.95 (0.32–2.79)		11/10	1.06 (0.43–2.61)	
		GC/CC *vs.* GG	161/187	0.85 (0.62–1.16)	0.31	143/165	0.68 (0.48–0.96)	**0.03**	64/97	0.88 (0.61–1.26)	0.47	86/87	1.16 (0.81–1.67)	0.43
*ALOX5*	rs1051713	CC	227/251	1.00 (ref)	0.56	210/189	1.00 (ref)	0.32	204/276	1.00 (ref)	0.11	189/210	1.00 (ref)	0.58
		CT	90/89	1.18 (0.83–1.69)		83/91	0.84 (0.58–1.22)		76/97	1.07 (0.75–1.53)		98/96	1.17 (0.82–1.66)	
		TT	11/9	1.36 (0.54–3.41)		13/7	1.75 (0.64–4.75)		5/19	0.36 (0.13–0.97)		10/15	0.81 (0.34–1.90)	
		TT *vs.* CC/CT	317/340	1.30 (0.52–3.23)	0.57	293/280	1.84 (0.68–4.98)	0.23	280/373	0.35 (0.13–0.95)	**0.04**	287/306	0.77 (0.33–1.78)	0.54
*ALOX5AP*	rs4293222	GG	136/148	1.00 (ref)	0.89	135/129	1.00 (ref)	0.74	13/31	1.00 (ref)	0.27	28/13	1.00 (ref)	0.09
		GC	148/150	1.06 (0.76–1.48)		127/126	0.94 (0.65–1.36)		104/148	1.56 (0.77–3.15)		115/128	0.46 (0.22–0.96)	
		CC	47/52	0.95 (0.59–1.52)		44/33	1.17 (0.68–2.01)		168/214	1.75 (0.87–3.50)		154/181	0.45 (0.22–0.92)	
		GC/CC *vs.* GG	195/202	1.03 (0.75–1.41)	0.87	171/159	0.99 (0.70–1.39)	0.96	272/362	1.66 (0.84–3.28)	0.14	269/309	0.45 (0.23–0.92)	**0.03**
*ALOX5AP*	rs9315045	TT	181/194	1.00 (ref)	0.94	174/162	1.00 (ref)	0.91	92/159	1.00 (ref)	0.10	92/101	1.00 (ref)	0.71
		TC	121/130	1.02 (0.73–1.42)		109/108	0.94 (0.66–1.35)		135/167	1.33 (0.94–1.88)		162/169	1.08 (0.75–1.56)	
		CC	28/25	1.11 (0.61–2.03)		23/18	1.07 (0.54–2.13)		59/63	1.57 (1.01–2.45)		43/51	0.89 (0.53–1.49)	
		TC/CC *vs.* TT	149/155	1.04 (0.76–1.42)	0.83	132/126	0.96 (0.68–1.35)	0.82	194/230	1.39 (1.01–1.93)	**0.04**	205/220	1.03 (0.73–1.47)	0.85
*ALOX5AP*	rs9315048	GG	185/211	1.00 (ref)	0.09	183/177	1.00 (ref)	0.93	124/198	1.00 (ref)	0.11	146/156	1.00 (ref)	0.90
		GT	120/125	1.12 (0.80–1.55)		100/95	1.07 (0.74–1.54)		124/160	1.22 (0.88–1.69)		121/134	0.93 (0.66–1.32)	
		TT	26/13	2.20 (1.08–4.51)		23/16	1.09 (0.54–2.21)		36/33	1.72 (1.02–2.91)		27/30	0.90 (0.50–1.62)	
		TT *vs.* GG/GT	305/336	2.11 (1.05–4.28)	**0.04**	283/272	1.07 (0.53–2.14)	0.85	248/358	1.56 (0.95–2.59)	0.08	267/290	0.93 (0.53–1.63)	0.80
*ALOX12*	rs2292350	GG	109/100	1.00 (ref)	0.56	89/107	1.00 (ref)	0.06	227/304	1.00 (ref)	0.41	235/238	1.00 (ref)	0.24
		GA	164/187	0.83 (0.58–1.18)		149/131	1.41 (0.95–2.07)		55/80	0.90 (0.61–1.34)		57/79	0.71 (0.47–1.06)	
		AA	57/61	0.84 (0.53–1.34)		67/49	1.76 (1.08–2.86)		3/9	0.42 (0.11–1.59)		5/5	1.02 (0.28–3.71)	
		GA/AA *vs.* GG	221/248	0.83 (0.59–1.16)	0.28	216/180	1.50 (1.04–2.16)	**0.03**	58/89	0.86 (0.58–1.26)	0.44	62/84	0.72 (0.49–1.08)	0.11
*ALOX12* ^e^	rs3840880^f^	TT	103/109	1.00 (ref)	0.67	118/78	1.00 (ref)	**0.006**	62/87	1.00 (ref)	0.56	71/72	1.00 (ref)	0.35
		TG	172/173	1.11 (0.78–1.58)		141/143	0.62 (0.42–0.92)		141/177	1.10 (0.74–1.64)		138/168	0.86 (0.57–1.30)	
		GG	54/64	0.92 (0.58–1.47)		47/67	0.47 (0.28–0.77)		82/129	0.90 (0.59–1.39)		89/82	1.15 (0.72–1.82)	
		TG/GG *vs.* TT	226/237	1.06 (0.76–1.48)	0.74	188/210	0.57 (0.40–0.83)	**0.003**	223/306	1.02 (0.70–1.48)	0.92	227/250	0.95 (0.65–1.40)	0.81

a OR, odds ratio; 95%CI, 95% confidence interval.

b Adjusted for age, history of breast cancer in a first-degree relative, and proportion of European ancestry.

c P-trend for genetic dose response determined by coding genotypes as having 0, 1, or 2 variant allele, which was subsequently analyzed as an ordinal variable.

d P for heterogeneity from dominant or recessive models.

e All significant p-values were further adjusted for multiple comparisons using Bonferroni correction, with P <0.002 (0.05/30) considered statistically significant.

f Several SNPs on the ALOX12 gene, rs3840880, rs1126667, rs434473, rs1042357, were found in high LD with rs3840880 (r^2^ >0.98) in White women, with a similar association pattern (**Supplemental Table 2**). Bold, significant P-values.

Associations of each SNP and risk of ER positive (ER+) and ER negative (ER−) breast cancer were examined separately (**Supplemental Table S3**). Several associations were suggested to be specifically stronger and significant for ER+ or ER− breast cancer in either White or Black women (**Table 4**). In White women, an increased risk of ER+ breast cancer was observed for *COX2*-rs689470 (OR: 2.60, 95% CI: 1.46–4.63) in the dominant model, whereas a reduced risk was observed for *ALOX5*-rs7099874 (OR: 0.66, 95% CI: 0.51–0.85) in a dominant model. *ALOX5AP-*rs9315048 was associated with increased risk of ER− breast cancer (OR: 2.56, 95% CI: 1.25–5.25) in the recessive model. Among Black women, an increased risk of ER+ cancer was observed for *ALOX5*-rs1369214 (OR: 1.53, 95% CI: 1.15–2.03) and *ALOX5AP-*rs9579648 (OR: 2.36, 95% CI: 1.15–4.84) in the recessive models. A reduced risk was also observed for *COX2*-rs2745557 (OR: 0.64, 95% CI: 0.43–0.96) with risk of ER− breast cancer in the dominant model. In addition, although there was no significant association observed in either White or Black women, *COX2*-rs5275 was associated with increased risk among post-menopausal Black women and specifically a significant increased risk for ER− breast cancer (OR: 1.54, 95% CI: 1.08–2.19) in a recessive model. In contrast, there was an indication of inverse association for this SNP among post-menopausal White women (OR=0.69, 95% CI: 0.40–1.19) (**Supplemental Table S2**). The four *ALOX12-*SNPs that showed significant associations in White women were found to be associated with stronger reduced risk for ER− breast cancer, as shown for rs3840880 (OR: 0.62, 95% CI: 0.40–0.96).

**Table 4:** SNPs inflammation-related pathways and risk of breast cancer by ER status in the WCHS^a^.

Gene	SNP	Genotype	White						Black					
			Estrogen Receptor Positive			Estrogen Receptor Negative			Estrogen Receptor Positive			Estrogen Receptor Negative		
			# Case/Control	OR (95%CI)^b, c^	*P* ^d, e, f^	# Case/Control	OR (95% CI) ^b, c^	*P* ^d, e, f^	# Case/Control	OR (95% CI) ^b, c^	*P* ^d, e, f^	# Case/Control	OR (95% CI) ^b, c^	*P* ^d, e, f^
*COX2*	rs689470	CC	410/618	1.00 (ref)	**0.001**	99/618	1.00 (ref)	0.83	131/262	1.00 (ref)	0.61	51/262	1.00 (ref)	0.46
		CT	39/17	3.05 (1.66–5.59)		5/17	1.41 (0.48–4.19)		168/325	1.05 (0.78–1.39)		77/325	1.18 (0.80–1.75)	
		TT	1/3	0.08 (0.00–3.11)		0/3			52/127	0.86 (0.58–1.28)		35/127	1.36 (0.83–2.21)	
		CT/TT *vs.* CC	40/20	2.60 (1.46–4.63)	**0.001**	5/20	1.17 (0.39–3.50)	0.78	220/452	1.00 (0.76–1.31)	0.84	112/452	1.23 (0.85–1.78)	0.28
*COX2*	rs5275	TT	179/279	1.00 (ref)	0.70	46/279	1.00 (ref)	0.90	62/119	1.00 (ref)	0.76	23/119	1.00 (ref)	0.06
		TC	214/288	1.11 (0.85–1.45)		47/288	0.96 (0.62–1.50)		175/337	0.97 (0.67–1.39)		65/337	0.98 (0.58–1.66)	
		CC	54/70	1.14 (0.75–1.72)		11/70	0.84 (0.41–1.74)		110/251	0.88 (0.60–1.30)		74/251	1.52 (0.90–2.56)	
		CC *vs.* TT/TC	393/567	1.08 (0.73–1.59)	0.71	93/567	0.86 (0.49–1.88)	0.66	237/456	0.90 (0.68–1.19)	0.47	88/456	1.54 (1.08–2.19)	**0.02**
*COX2*	rs2745557	GG	288/397	1.00 (ref)	0.51	68/397	1.00 (ref)	0.42	249/503	1.00 (ref)	0.93	130/503	1.00 (ref)	0.10
		GA	142/203	0.97 (0.74–1.28)		27/203	0.82 (0.51–1.33)		91/190	0.96 (0.71–1.29)		32/190	0.63 (0.41–0.96)	
		AA	19/37	0.70 (0.39–1.27)		9/37	1.43 (0.65–3.12)		10/21	1.10 (0.50–2.39)		4/21	0.77 (0.26–2.29)	
		GA/AA *vs.* GG	161/240	0.93 (0.72–1.21)	0.59	36/240	0.92 (0.59–1.43)	0.71	101/211	0.97 (0.73–1.29)	0.84	36/211	0.64 (0.43–0.96)	**0.03**
*ALOX5*	rs1369214	GG	141/190	1.00 (ref)	0.53	30/190	1.00 (ref)	0.92	70/156	1.00 (ref)	**0.01**	30/156	1.00 (ref)	0.20
		GA	226/310	1.03 (0.77–1.37)		51/310	1.06 (0.65–1.73)		157/367	0.96 (0.68–1.36)		82/367	1.15 (0.73–1.83)	
		AA	80/134	0.85 (0.59–1.22)		23/134	1.13 (0.62–2.04)		122/189	1.49 (1.03–2.15)		54/189	1.53 (0.93–2.51)	
		AA *vs.* GG/GA	367/500	0.83 (0.60–1.15)	0.26	81/500	1.09 (0.66–1.81)	0.74	227/523	1.53 (1.15–2.03)	**0.003**	112/523	1.38 (0.95–1.99)	0.09
*ALOX5*	rs7099874	GG	246/281	1.00 (ref)	**0.001**	40/281	1.00 (ref)	0.44	248/525	1.00 (ref)	0.56	131/525	1.00 (ref)	0.24
		GC	160/304	0.62 (0.47–0.80)		52/304	1.20 (0.77–1.88)		91/165	1.16 (0.86–1.57)		28/165	0.70 (0.45–1.09)	
		CC	40/48	0.96 (0.60–1.54)		11/48	1.59 (0.75–3.35)		9/19	0.84 (0.37–1.92)		6/19	1.26 (0.49–3.25)	
		GC/CC *vs.* GG	200/352	0.66 (0.51–0.85)	**0.001**	63/352	1.26 (0.82–1.93)	0.30	100/184	1.12 (0.84–1.50)	0.44	34/184	0.76 (0.50–1.15)	0.19
*ALOX5AP*	rs9579648	GG	319/441	1.00 (ref)	0.66	69/441	1.00 (ref)	0.82	235/473	1.00 (ref)	0.06	114/473	1.00 (ref)	0.74
		GC	121/177	0.94 (0.71–1.25)		31/177	1.08 (0.68–1.72)		99/217	0.97 (0.72–1.29)		47/217	0.92 (0.63–1.34)	
		CC	8/19	0.69 (0.29–1.62)		4/19	1.38 (0.45–4.20)		16/16	2.33 (1.13–4.82)		5/16	1.36 (0.49–3.82)	
		CC *vs.* GG/GC	440/618	0.70 (0.30–1.64)	0.42	100/618	1.35 (0.45–4.06)	0.60	334/690	2.36 (1.15–4.84)	**0.02**	161/690	1. 40 (0.50–3.90)	0.52
*ALOX5AP*	rs9315048	GG	256/388	1.00 (ref)	0.26	62/388	1.00 (ref)	**0.03**	173/354	1.00 (ref)	0.76	72/354	1.00 (ref)	0.37
		GT	164/220	1.17 (0.90–1.52)		30/220	0.90 (0.56–1.45)		141/294	0.96 (0.73–1.26)		70/294	1.15 (0.79–1.65)	
		TT	30/29	1.47 (0.84–2.55)		12/29	2.47 (1.19–5.15)		36/63	1.14 (0.73–1.80)		20/63	1.49 (0.85–2.63)	
		TT *vs.* GG/GT	420/608	1.38 (0.80–2.38)	0.24	92/608	2.56 (1.25–5.25)	**0.01**	314/648	1.17 (0.75–1.81)	0.49	142/648	1.40 (0.82–2.39)	0.22
*ALOX12*	rs3840880^g^	TT	154/187	1.00 (ref)	0.27	43/187	1.00 (ref)	**0.01**	79/159	1.00 (ref)	0.20	39/159	1.00 (ref)	0.07
		TG	217/316	0.87 (0.65–1.15)		51/316	0.75 (0.47–1.17)		154/345	0.90 (0.64–1.26)		93/345	1.05 (0.69–1.60)	
		GG	78/131	0.74 (0.52–1.07)		10/131	0.34 (0.16–0.70)		117/211	1.19 (0.83–1.70)		34/211	0.63 (0.38–1.06)	
		TG/GG *vs.* TT	295/447	0.83 (0.63–1.09)	0.18	61/447	0.62 (0.40–0.96)	**0.03**	271/556	1.00 (0.73–1.37)	0.99	127/556	0.89 (0.60–1.34)	0.59

a Based 554 (87.0%) White cases and 517 (88.5%) Black cases with available data on ER status.

b OR, odds ratio; 95%CI, 95% confidence interval.

c Adjusted for age, family history of breast cancer in a first-degree relative, and proportion of European ancestry.

d P-trend for genetic dose response determined by coding genotypes as having 0, 1, or 2 variant allele, which was subsequently analyzed as an ordinal variable.

e P for heterogeneity from dominant or recessive models.

f All significant p-values were further adjusted for multiple comparisons using Bonferroni correction, with P <0.002 (0.05/30) considered statistically significant.

g Several SNPs on the ALOX12 gene, rs3840880, rs1126667, rs434473, rs1042357, were found in high LD with rs3840880 (r^2^ >0.98) in White women, with a similar association pattern (**Supplemental Table 3**).

## Discussion

In this large case-control study of White and Black women, we examined candidate genetic variants in four genes involved in *COX-2* and *ALOX* inflammation-related pathways and risk of breast cancer. Allele frequencies of some of the SNPs in these genes varied significantly between White and Black populations. A number of these SNPs in *COX2*, *ALOX5*, *ALOX5AP*, and *ALOX12* genes were found to be associated with overall breast cancer risk, as well as breast cancer risk in subgroups defined by menopausal and ER status, in either White or Black women. To our knowledge, this is among the first study to examine associations of genetic variants of these genes with breast cancer within and across White and Black populations, specifically in a large number of Black women.

The *COX2*–prostaglandin pathway links inflammation and tumorigenesis by providing a tumor-promoting microenvironment (13). Elevated levels of *COX-2* and its metabolites, such as PGE2, an inflammatory mediator, have been associated with aggressive breast cancer phenotypes and poor survival (13, 30, 31), whereas inhibition of *COX-2* activity has shown anti-tumor and therapeutic effects in preclinical models and population studies (12, 32–35). Particular attention has been given to the influence of rs5275, which is located in the 3′ untranslated region (3’UTR) and is among the most common *COX-2* polymorphisms in White women (36). Associations for rs5275 with breast cancer have been inconsistent; studies focusing on Whites in a US population suggested a reduced risk for TT (37) or CC genotypes (36, 38), whereas one study in a Brazilian population reported an increased risk for the heterozygous TC genotype (39) and others reported no significant association (40–42). In our study, rs5275 was not associated with overall breast cancer risk in either White or Black women, but a significant increased risk for ER− cancer was observed for the CC genotype among Black women, while there was a suggestive reduced risk among White postmenopausal women. *COX2-*rs689470 was significantly associated with increased risk for breast cancer in White women, with stronger associations among those who were post-menopausal and for ER+ breast cancer. Compare to our study, an earlier small study involving 180 breast cancer cases in postmenopausal women, however, failed to observe a significant association (43). Both SNPs are located in the 3’UTR and may contribute to breast carcinogenesis through transcriptional or post-transcriptional regulation of *COX2* expression. Among Black women, rs2745557 was associated with decreased risk, specifically among postmenopausal women and for ER− breast cancer. This SNP has been linked to an increased breast cancer risk among primarily White populations in previous studies (36, 44). Our above observations, coupled with existing evidence that *COX-2* levels vary by menopausal and ER status (45–47), suggest a potential menopausal/estrogen-mediated role in the *COX2*–prostaglandin-carcinogenesis pathway. Previous studies have been mostly limited to White populations and have not considered menopausal or ER status, which may explain the inconsistent findings in studies of these SNPs in relation to breast cancer (39, 40). Further studies are needed to confirm the interrelationships of *COX2* genetic variants, menopausal status, tumor subtypes, and ancestry.

Like the COX pathway, studies have shown that 5-lipoxygenase (5LOX) and its metabolites are upregulated in multiple cancers and play a potential role in carcinogenesis (48). In this study, we identified several SNPs in the *ALOX5* and *ALOX5AP* genes that showed associations with overall breast cancer risk and differences by menopausal and ER status, in either White or Black women. The *ALOX5*-rs7099874 was associated with reduced risk in postmenopausal White women. Significant associations were observed for two *ALOX5 SNPs* (rs1369214 and rs1051713) and the *ALOX5AP*-rs9315045 in premenopausal Black women, and *ALOX5AP*-rs4293222 in postmenopausal Black women. The *ALOX5*-rs7099874 and *ALOX5AP*-rs9579648 were specifically associated risk of ER+ breast cancer, while *ALOX5AP*-rs9315048 were associated with risk of ER− cancer, in either White or Black women. These SNPs in relation to breast cancer were not well studied or reported in the literature, our findings provide some evidence of their potential role in breast cancer susceptibility and potential differences by menopausal and ER status.

ALOX12 and its metabolite, 12S-hydroxyeicosatetraenoic acid, have been implicated in influencing tumor transformation and progression (49, 50). A recent study showed an upregulation of ALOX12 in breast cancer cell lines and tumor tissues compared to their corresponding normal breast cells and tissues (51). We identified a group of SNPs (rs3840880, rs1126667, rs434473, rs1042357) that were found in high LD in Whites, but not in Blacks. Interestingly, we observed that these SNPs were significantly associated with overall breast cancer risk, specifically in postmenopausal White women only, and the associated reduced risk was stronger for ER− breast cancer. In addition, there is some evidence that the presence of minor alleles of the rs434473 was associated with early onset of natural menopause in White women (52), which may explain the lower breast cancer risk observed among these women. A previous study, however, suggested that rs434473 was associated with an increased risk, especially among women with regular nonsteroidal anti-inflammatory drug use (21). We also observed an increased risk of breast cancer with the major alleles of rs2292350 among post-menopausal White women. An association between this SNP and age at menopause has also been found in a population of Chinese women (53). Frequencies of these polymorphisms have been found to differ across populations, and future studies are needed to confirm these associations.

Several limitations warrant consideration. First, we selected a panel of candidate SNPs based on their potential functions from previous studies, other potentially important genetic variants may not be included in the current study. However, analysis of these common genetic variants in candidate pathways is a more focused method for increasing our knowledge of potentially important biological pathways in the etiology of breast cancer (54). Second, although this is a study with a large number of White and Black women, which allow us to examine racial differences for these genetic variants with breast cancer risk, our sample size was relatively limited when analyses were stratified by menopausal and ER status. Third, the majority of associations did not reach statistical significance after Bonferroni corrections for multiple comparisons, thus we cannot exclude the possibility of false positive findings. Lastly, as the SNP-breast cancer associations may be confounded by other potential risk factors, we examined whether inclusion of these factors as covariates change the risk estimate, and subsequently presented results from the multivariable-adjusted model to minimize residual confounding. Nevertheless, this study investigates the role of *COX* and *LOX* genetic variants in a large number of Black women, which addresses the unmet need to improve representation of Black populations in genomic breast cancer studies. Furthermore, SNP associations with breast cancer risk were found to differ between White and Black populations, especially when menopausal and ER statuses were considered. These findings may provide insights into the etiology of breast cancer, indicating areas for further research into reasons for breast cancer racial differences.

In conclusion, this study is among the first to examine genetic variants in genes involved in the COX- and LOX-related inflammatory pathways with breast cancer risk among both White and Black women. Our study suggests that genetic variants of these inflammation-related genes are associated with breast cancer, and that these associations and genotype distributions differ in subgroups defined by menopausal and ER status between White and Black women. As current research remains limited, additional studies are necessary to confirm these findings and explore the underlying molecular mechanisms.

## Data Availability Statement

The datasets presented in this study can be found in online repositories. The names of the repository/repositories and accession number(s) can be found in the article/**Supplementary Material**.

## Ethics Statement

This study was approved by institutional review boards at the Roswell Park Comprehensive Cancer Center, the Rutgers Cancer Institute of New Jersey, Mount Sinai School of Medicine (now the Icahn School of Medicine at Mount Sinai), and participating hospitals in New York City. Signed informed consent was obtained from each participant prior to interview and biospecimen collection. The patients/participants provided their written informed consent to participate in this study.

## Author Contributions

JM made substantial contributions to the interpretation of the data, drafted the manuscript, and revised it critically for important intellectual content. CA, EB, CCH, AO, GZ, TK and SY were essential to data collection and genotyping, interpretation of the data and manuscript writing. ZG made substantial contributions to the conception, design, analysis, and writing the manuscript. All authors contributed to the article and approved the submitted version.

## Funding

This work was supported by grants from the National Cancer Institute (T32CA113951, K07 CA178293, R01 CA100598, P01 CA151135, P30CA072720, P30 CA016056), the Breast Cancer Research Foundation and a gift from the Philip L. Hubbell Family. The New Jersey State Cancer Registry (NJSCR) is a participant in the Centers for Disease Control and Prevention’s National Program of Cancer Registries and is a National Cancer Institute Surveillance, Epidemiology, and End Results (SEER) Expansion Registry. The NJSCR is supported by the Centers for Disease Control and Prevention under cooperative agreement DP07-703 awarded to the New Jersey Department of Health & Senior Services. The collection of New Jersey cancer incidence data is also supported by the National Cancer Institute’s SEER Program under contract N01-PC-95001-20 and the State of New Jersey. The funders had no role in study design, data collection and analysis, decision to publish, or preparation of the manuscript.

## Conflict of Interest

The authors declare that the research was conducted in the absence of any commercial or financial relationships that could be construed as a potential conflict of interest.
