# Supplementary material for: Genetic Variants in COX2 and ALOX Genes and Breast Cancer Risk in White and Black Women
Source: Front Oncol. 2021 Jun 24;11:679998. doi: 10.3389/fonc.2021.679998 (PMC8263909; doi:10.3389/fonc.2021.679998)
Supplement: Supplementary file 1 [file Table_1.docx]

**Supplemental Table S1. SNPs of inflammation-related pathways and risk of breast cancer among white and Black women in the WCHS**

| **Gene** | **SNP** | **Chr** | **Coordinate** | **Genotype** | **White** | | | | **Black** | | | | **Interaction** |
| --- | --- | --- | --- | --- | --- | --- | --- | --- | --- | --- | --- | --- | --- |
|  |  |  |  |  | **#Case/Control** | **OR (95% CI)^a^** | **OR (95% CI)^b,c^** | ***P*^d,e,f^** | **#Case/Control** | **OR (95% CI)^a^** | **OR (95% CI)^b,c^** | **P^d,e,f^** | ***P*^g^** |
| COX2 | rs689470 | 1 | 186641058 | CC | 589/618 | 1.00 (ref) | 1.00 (ref) | **0.004** | 205/262 | 1.00 (ref) | 1.00 (ref) | 0.86 | **0.006** |
|  |  |  |  | CT | 47/17 | 2.84 (1.60-5.03) | 2.37 (1.32-4.27) |  | 273/325 | 1.06 (0.83-1.36) | 1.07 (0.83-1.38) |  |  |
|  |  |  |  | TT | 1/3 | 0.42 (0.04-4.06) | 0.05 (0.00-2.19) |  | 102/127 | 1.06 (0.77-1.46) | 1.06 (0.77-1.48) |  |  |
|  |  |  |  | Per copy variant allele |  | 2.06 (1.26-3.36) | 1.67 (1.00-2.79) | 0.05 |  | 1.03 (0.89-1.21) | 1.04 (0.88-1.22) | 0.65 |  |
|  |  |  |  | CT/TT vs. CC | 48/20 | 2.49 (1.45-4.28) | 2.02 (1.16-3.53) | **0.01** | 375/452 | 1.06 (0.84-1.34) | 1.07 (0.84-1.35) | 0.58 |  |
|  |  |  |  | TT vs. CC/CT | 636/635 | 0.40 (0.04-3.88) | 0.05 (0.00-2.19) | 0.12 | 478/587 | 1.03 (0.77-1.37) | 1.02 (0.76-1.37) | 0.89 |  |
| COX2 | rs2206593 | 1 | 186642429 | GG | 550/542 | 1.00 (ref) | 1.00 (ref) | 0.51 | 577/701 | 1.00 (ref) | 1.00 (ref) | 0.18 | 0.36 |
|  |  |  |  | GA | 78/89 | 0.86 (0.62-1.19) | 0.86 (0.62-1.21) |  | 5/13 | 0.50 (0.18-1.42) | 0.49 (0.17-1.40) |  |  |
|  |  |  |  | AA | 6/4 | 1.57 (0.44-5.64) | 1.65 (0.46-5.95) |  | 0/0 |  |  |  |  |
|  |  |  |  | Per copy variant allele |  | 0.93 (0.69-1.25) | 0.94 (0.70-1.27) | 0.68 |  | 0.50 (0.18-1.42) | 0.49 (0.17-1.40) | 0.18 |  |
|  |  |  |  | GA/AA vs. GG | 84/93 | 0.89 (0.64-1.22) | 0.90 (0.65-1.24) | 0.51 | 5/13 | 0.50 (0.18-1.42) | 0.49 (0.17-1.40) | 0.18 |  |
|  |  |  |  | AA vs. GG/GA | 628/631 | 1.60 (0.45-5.76) | 1.68 (0.46-6.07) | 0.43 |  |  |  |  |  |
| COX2 | rs5275 | 1 | 186643058 | TT | 269/279 | 1.00 (ref) | 1.00 (ref) | 0.96 | 92/119 | 1.00 (ref) | 1.00 (ref) | 0.68 | 0.89 |
|  |  |  |  | TC | 293/288 | 1.05 (0.83-1.32) | 1.02 (0.80-1.30) |  | 271/337 | 1.01 (0.73-1.39) | 1.01 (0.73-1.39) |  |  |
|  |  |  |  | CC | 71/70 | 1.01 (0.70-1.47) | 0.97 (0.66-1.42) |  | 213/251 | 1.12 (0.80-1.56) | 1.12 (0.80-1.56) |  |  |
|  |  |  |  | Per copy variant allele |  | 1.02 (0.86-1.21) | 1.00 (0.84-1.18) | 0.98 |  | 1.07 (0.91-1.25) | 1.07 (0.91-1.25) | 0.43 |  |
|  |  |  |  | TC/CC vs. TT | 364/358 | 1.04 (0.83-1.30) | 1.01 (0.81-1.27) | 0.92 | 484/588 | 1.06 (0.78-1.43) | 1.05 (0.78-1.43) | 0.73 |  |
|  |  |  |  | CC vs. TT/TC | 562/567 | 0.99 (0.70-1.41) | 0.96 (0.67-1.38) | 0.83 | 363/456 | 1.11 (0.88-1.40) | 1.11 (0.88-1.41) | 0.38 |  |
| COX2 | rs4648274 | 1 | 186646480 | AA | 631/633 | 1.00 (ref) | 1.00 (ref) | 0.17 | 439/541 | 1.00 (ref) | 1.00 (ref) | 0.76 | 0.81 |
|  |  |  |  | AC | 4/4 | 1.07 (0.26-4.37) | 0.29 (0.05-1.69) |  | 129/160 | 1.01 (0.77-1.32) | 1.02 (0.78-1.33) |  |  |
|  |  |  |  | CC | 0/0 |  |  |  | 12/12 | 1.38 (0.61-3.15) | 1.36 (0.60-3.12) |  |  |
|  |  |  |  | Per copy variant allele |  | 1.07 (0.26-4.37) | 0.29 (0.05-1.69) | 0.17 |  | 1.05 (0.84-1.33) | 1.06 (0.84-1.33) | 0.65 |  |
|  |  |  |  | AC/CC vs. AA | 4/4 | 1.07 (0.26-4.37) | 0.29 (0.05-1.69) | 0.17 | 141/172 | 1.04 (0.80-1.34) | 1.04 (0.80-1.35) | 0.78 |  |
|  |  |  |  | CC vs. AA/AC |  |  |  |  | 568/701 | 1.38 (0.61-3.14) | 1.36 (0.60-3.10) | 0.47 |  |
| COX2 | rs2745557 | 1 | 186649221 | GG | 404/397 | 1.00 (ref) | 1.00 (ref) | 0.82 | 430/503 | 1.00 (ref) | 1.00 (ref) | 0.40 | 0.73 |
|  |  |  |  | GA | 197/203 | 0.94 (0.74-1.20) | 0.98 (0.77-1.26) |  | 137/190 | 0.84 (0.65-1.08) | 0.84 (0.65-1.08) |  |  |
|  |  |  |  | AA | 34/37 | 0.91 (0.55-1.48) | 0.85 (0.51-1.40) |  | 16/21 | 0.95 (0.49-1.86) | 0.96 (0.49-1.88) |  |  |
|  |  |  |  | Per copy variant allele |  | 0.95 (0.79-1.14) | 0.95 (0.79-1.15) | 0.61 |  | 0.88 (0.71-1.10) | 0.89 (0.71-1.1) | 0.27 |  |
|  |  |  |  | GA/AA vs. GG | 231/240 | 0.94 (0.75-1.18) | 0.96 (0.76-1.21) | 0.74 | 153/211 | 0.85 (0.66-1.09) | 0.85 (0.66-1.09) | 0.20 |  |
|  |  |  |  | AA vs. GG/GA | 601/600 | 0.92 (0.57-1.50) | 0.86 (0.52-1.40) | 0.54 | 567/693 | 1.00 (0.51-1.94) | 1.01 (0.52-1.97) | 0.98 |  |
| COX2 | rs689466 | 1 | 186650751 | AA | 428/451 | 1.00 (ref) | 1.00 (ref) | 0.23 | 479/582 | 1.00 (ref) | 1.00 (ref) | 0.87 | 0.50 |
|  |  |  |  | AG | 183/169 | 1.17 (0.91-1.50) | 1.19 (0.92-1.53) |  | 96/124 | 0.92 (0.68-1.24) | 0.92 (0.68-1.24) |  |  |
|  |  |  |  | GG | 22/16 | 1.46 (0.75-2.83) | 1.51 (0.77-2.97) |  | 6/7 | 0.98 (0.32-2.99) | 0.96 (0.31-2.93) |  |  |
|  |  |  |  | Per copy variant allele |  | 1.18 (0.96-1.46) | 1.20 (0.97-1.49) | 0.09 |  | 0.93 (0.72-1.22) | 0.93 (0.71-1.22) | 0.61 |  |
|  |  |  |  | AG/GG vs. AA | 205/185 | 1.20 (0.94-1.52) | 1.22 (0.95-1.56) | 0.12 | 102/131 | 0.92 (0.69-1.23) | 0.92 (0.69-1.24) | 0.59 |  |
|  |  |  |  | GG vs. AA/AG | 611/620 | 1.39 (0.72-2.69) | 1.44 (0.74-2.81) | 0.29 | 575/706 | 1.00 (0.33-3.03) | 0.97 (0.32-2.97) | 0.96 |  |
| ALOX5 | rs6593482 | 10 | 45868449 | GG | 437/454 | 1.00 (ref) | 1.00 (ref) | 0.24 | 396/492 | 1.00 (ref) | 1.00 (ref) | 0.93 | 0.41 |
|  |  |  |  | GT | 172/164 | 1.09 (0.84-1.40) | 1.15 (0.89-1.49) |  | 164/191 | 1.04 (0.81-1.34) | 1.04 (0.81-1.33) |  |  |
|  |  |  |  | TT | 22/15 | 1.71 (0.87-3.37) | 1.66 (0.83-3.32) |  | 18/23 | 0.93 (0.49-1.76) | 0.93 (0.49-1.76) |  |  |
|  |  |  |  | Per copy variant allele |  | 1.16 (0.94-1.43) | 1.19 (0.96-1.48) | 0.11 |  | 1.01 (0.82-1.24) | 1.01 (0.82-1.24) | 0.92 |  |
|  |  |  |  | GT/TT vs. GG | 194/179 | 1.14 (0.89-1.45) | 1.19 (0.93-1.53) | 0.17 | 182/214 | 1.03 (0.81-1.31) | 1.03 (0.81-1.31) | 0.83 |  |
|  |  |  |  | TT vs. GG/GT | 609/618 | 1.67 (0.85-3.28) | 1.60 (0.80-3.18) | 0.18 | 560/683 | 0.92 (0.49-1.73) | 0.92 (0.49-1.74) | 0.80 |  |
| ALOX5 | rs7099684 | 10 | 45897155 | TT | 396/385 | 1.00 (ref) | 1.00 (ref) | 0.41 | 470/552 | 1.00 (ref) | 1.00 (ref) | 0.27 | 0.54 |
|  |  |  |  | TA | 212/229 | 0.89 (0.71-1.13) | 0.87 (0.68-1.10) |  | 106/150 | 0.82 (0.62-1.09) | 0.82 (0.62-1.09) |  |  |
|  |  |  |  | AA | 28/24 | 1.10 (0.62-1.94) | 1.14 (0.64-2.04) |  | 6/12 | 0.64 (0.23-1.73) | 0.61 (0.22-1.66) |  |  |
|  |  |  |  | Per copy variant allele |  | 0.95 (0.78-1.15) | 0.94 (0.77-1.15) | 0.55 |  | 0.82 (0.64-1.05) | 0.81 (0.63-1.04) | 0.11 |  |
|  |  |  |  | TA/AA vs. TT | 240/253 | 0.91 (0.73-1.15) | 0.89 (0.71-1.13) | 0.34 | 112/162 | 0.81 (0.61-1.06) | 0.81 (0.61-1.06) | 0.13 |  |
|  |  |  |  | AA vs. TT/TA | 608/614 | 1.15 (0.65-2.01) | 1.20 (0.68-2.13) | 0.53 | 576/702 | 0.66 (0.24-1.79) | 0.63 (0.23-1.71) | 0.37 |  |
| ALOX5 | rs1369214 | 10 | 45900729 | GG | 195/190 | 1.00 (ref) | 1.00 (ref) | 0.83 | 112/156 | 1.00 (ref) | 1.00 (ref) | **0.01** | 0.08 |
|  |  |  |  | GA | 316/310 | 1.01 (0.78-1.31) | 1.03 (0.79-1.33) |  | 274/367 | 1.04 (0.77-1.39) | 1.04 (0.78-1.40) |  |  |
|  |  |  |  | AA | 123/134 | 0.95 (0.69-1.31) | 0.94 (0.68-1.30) |  | 196/189 | 1.48 (1.07-2.03) | 1.48 (1.08-2.04) |  |  |
|  |  |  |  | Per copy variant allele |  | 0.98 (0.84-1.15) | 0.97 (0.83-1.14) | 0.74 |  | 1.23 (1.05-1.45) | 1.24 (1.06-1.45) | **0.008** |  |
|  |  |  |  | GA/AA vs. GG | 439/444 | 1.00 (0.78-1.27) | 1.00 (0.78-1.28) | 1.00 | 470/556 | 1.18 (0.9-1.56) | 1.19 (0.90-1.57) | 0.22 |  |
|  |  |  |  | AA vs. GG/GA | 511/500 | 0.94 (0.71-1.24) | 0.92 (0.69-1.22) | 0.57 | 386/523 | 1.44 (1.13-1.83) | 1.44 (1.13-1.84) | **0.003** |  |
| ALOX5 | rs2228065 | 10 | 45920506 | GG | 632/636 | 1.00 (ref) | 1.00 (ref) | 0.39 | 494/605 | 1.00 (ref) | 1.00 (ref) | 0.98 | 0.70 |
|  |  |  |  | GA | 3/2 | 1.76 (0.29-10.71) | 0.29 (0.02-4.79) |  | 85/104 | 0.97 (0.71-1.32) | 0.97 (0.71-1.33) |  |  |
|  |  |  |  | AA | 0/0 |  |  |  | 4/6 | 0.94 (0.26-3.41) | 0.95 (0.26-3.44) |  |  |
|  |  |  |  | Per copy variant allele |  | 1.76 (0.29-10.71) | 0.29 (0.02-4.79) | 0.39 |  | 0.97 (0.73-1.29) | 0.97 (0.73-1.30) | 0.85 |  |
|  |  |  |  | GA/AA vs. GG | 3/2 | 1.76 (0.29-10.71) | 0.29 (0.02-4.79) | 0.39 | 89/110 | 0.96 (0.71-1.31) | 0.97 (0.71-1.32) | 0.86 |  |
|  |  |  |  | AA vs. GG/GA |  |  |  |  | 579/709 | 0.95 (0.26-3.42) | 0.95 (0.26-3.45) | 0.94 |  |
| ALOX5 | rs1487562 | 10 | 45928822 | CC | 419/428 | 1.00 (ref) | 1.00 (ref) | 0.13 | 337/400 | 1.00 (ref) | 1.00 (ref) | 0.33 | **0.03** |
|  |  |  |  | CT | 180/190 | 0.97 (0.76-1.24) | 1.01 (0.78-1.29) |  | 211/258 | 0.97 (0.77-1.23) | 0.97 (0.77-1.23) |  |  |
|  |  |  |  | TT | 37/20 | 1.90 (1.08-3.35) | 1.80 (1.02-3.20) |  | 34/57 | 0.71 (0.45-1.12) | 0.71 (0.45-1.12) |  |  |
|  |  |  |  | Per copy variant allele |  | 1.13 (0.93-1.37) | 1.14 (0.93-1.39) | 0.20 |  | 0.90 (0.75-1.08) | 0.90 (0.75-1.08) | 0.25 |  |
|  |  |  |  | CT/TT vs. CC | 217/210 | 1.06 (0.84-1.34) | 1.08 (0.85-1.38) | 0.51 | 245/315 | 0.92 (0.74-1.15) | 0.92 (0.74-1.15) | 0.48 |  |
|  |  |  |  | TT vs. CC/CT | 599/618 | 1.92 (1.10-3.37) | 1.80 (1.02-3.18) | **0.04** | 548/658 | 0.72 (0.46-1.12) | 0.72 (0.46-1.12) | 0.14 |  |
| ALOX5 | rs7099874 | 10 | 45928911 | GG | 328/281 | 1.00 (ref) | 1.00 (ref) | **0.02** | 429/525 | 1.00 (ref) | 1.00 (ref) | 0.99 | 0.15 |
|  |  |  |  | GC | 249/304 | 0.70 (0.55-0.88) | 0.71 (0.56-0.9) |  | 133/165 | 0.97 (0.75-1.27) | 0.98 (0.75-1.28) |  |  |
|  |  |  |  | CC | 55/48 | 0.96 (0.63-1.47) | 0.98 (0.64-1.51) |  | 17/19 | 1.01 (0.51-1.99) | 1.00 (0.51-1.98) |  |  |
|  |  |  |  | Per copy variant allele |  | 0.85 (0.71-1.01) | 0.86 (0.72-1.03) | 0.09 |  | 0.98 (0.79-1.22) | 0.99 (0.79-1.23) | 0.91 |  |
|  |  |  |  | GC/CC vs. GG | 304/352 | 0.74 (0.59-0.92) | 0.75 (0.60-0.94) | **0.01** | 150/184 | 0.98 (0.76-1.26) | 0.98 (0.76-1.27) | 0.89 |  |
|  |  |  |  | CC vs. GG/GC | 577/585 | 1.14 (0.76-1.72) | 1.16 (0.76-1.75) | 0.49 | 562/690 | 1.02 (0.52-1.99) | 1.01 (0.51-1.99) | 0.98 |  |
| ALOX5 | rs1051713 | 10 | 45938746 | CC | 437/440 | 1.00 (ref) | 1.00 (ref) | 0.41 | 393/486 | 1.00 (ref) | 1.00 (ref) | 0.10 | 0.07 |
|  |  |  |  | CT | 173/180 | 0.97 (0.76-1.25) | 0.98 (0.76-1.26) |  | 174/193 | 1.08 (0.85-1.39) | 1.10 (0.85-1.40) |  |  |
|  |  |  |  | TT | 24/16 | 1.48 (0.77-2.84) | 1.54 (0.80-2.98) |  | 15/34 | 0.53 (0.28-1.00) | 0.54 (0.29-1.02) |  |  |
|  |  |  |  | Per copy variant allele |  | 1.05 (0.86-1.30) | 1.07 (0.86-1.32) | 0.55 |  | 0.93 (0.76-1.14) | 0.94 (0.77-1.15) | 0.54 |  |
|  |  |  |  | CT/TT vs. CC | 197/196 | 1.01 (0.80-1.29) | 1.02 (0.80-1.31) | 0.85 | 189/227 | 1.00 (0.79-1.27) | 1.01 (0.80-1.28) | 0.92 |  |
|  |  |  |  | TT vs. CC/CT | 610/620 | 1.49 (0.78-2.85) | 1.55 (0.81-2.99) | 0.19 | 567/679 | 0.52 (0.28-0.97) | 0.53 (0.28-0.98) | **0.04** |  |
| ALOX5AP | rs4076128 | 13 | 31305143 | AA | 325/317 | 1.00 (ref) | 1.00 (ref) | 0.76 | 38/44 | 1.00 (ref) | 1.00 (ref) | 0.90 | 0.83 |
|  |  |  |  | AG | 253/262 | 0.95 (0.75-1.20) | 0.92 (0.72-1.16) |  | 207/250 | 0.94 (0.58-1.52) | 0.94 (0.58-1.52) |  |  |
|  |  |  |  | GG | 56/55 | 0.99 (0.66-1.49) | 0.93 (0.61-1.41) |  | 335/417 | 0.91 (0.57-1.45) | 0.91 (0.56-1.46) |  |  |
|  |  |  |  | Per copy variant allele |  | 0.98 (0.82-1.16) | 0.94 (0.79-1.12) | 0.52 |  | 0.96 (0.80-1.15) | 0.96 (0.79-1.16) | 0.66 |  |
|  |  |  |  | AG/GG vs. AA | 309/317 | 0.96 (0.77-1.19) | 0.92 (0.73-1.15) | 0.46 | 542/667 | 0.92 (0.59-1.46) | 0.92 (0.58-1.46) | 0.73 |  |
|  |  |  |  | GG vs. AA/AG | 578/579 | 1.01 (0.68-1.5) | 0.96 (0.64-1.44) | 0.86 | 245/294 | 0.96 (0.76-1.20) | 0.96 (0.76-1.21) | 0.71 |  |
| ALOX5AP | rs4073259 | 13 | 31306271 | AA | 257/247 | 1.00 (ref) | 1.00 (ref) | 0.67 | 24/29 | 1.00 (ref) | 1.00 (ref) | 0.90 | 0.92 |
|  |  |  |  | AG | 279/293 | 0.92 (0.72-1.17) | 0.89 (0.70-1.14) |  | 176/222 | 0.92 (0.51-1.65) | 0.93 (0.52-1.68) |  |  |
|  |  |  |  | GG | 100/97 | 0.99 (0.71-1.38) | 0.94 (0.67-1.32) |  | 380/462 | 0.97 (0.55-1.72) | 0.99 (0.55-1.76) |  |  |
|  |  |  |  | Per copy variant allele |  | 0.98 (0.84-1.15) | 0.95 (0.81-1.12) | 0.54 |  | 1.03 (0.84-1.25) | 1.03 (0.84-1.26) | 0.77 |  |
|  |  |  |  | AG/GG vs. AA | 379/390 | 0.94 (0.75-1.18) | 0.90 (0.72-1.14) | 0.39 | 556/684 | 0.96 (0.54-1.68) | 0.96 (0.54-1.71) | 0.90 |  |
|  |  |  |  | GG vs. AA/AG | 536/540 | 1.04 (0.76-1.41) | 1.00 (0.73-1.36) | 0.98 | 200/251 | 1.05 (0.83-1.32) | 1.05 (0.83-1.34) | 0.69 |  |
| ALOX5AP | rs4293222 | 13 | 31311773 | GG | 271/277 | 1.00 (ref) | 1.00 (ref) | 0.97 | 41/44 | 1.00 (ref) | 1.00 (ref) | 0.75 | 0.54 |
|  |  |  |  | GC | 275/276 | 1.03 (0.81-1.31) | 1.02 (0.80-1.30) |  | 219/276 | 0.83 (0.52-1.33) | 0.84 (0.52-1.34) |  |  |
|  |  |  |  | CC | 91/85 | 1.10 (0.78-1.55) | 1.05 (0.74-1.49) |  | 322/395 | 0.87 (0.55-1.38) | 0.87 (0.55-1.39) |  |  |
|  |  |  |  | Per copy variant allele |  | 1.04 (0.89-1.22) | 1.02 (0.87-1.20) | 0.79 |  | 0.99 (0.82-1.18) | 0.99 (0.82-1.19) | 0.88 |  |
|  |  |  |  | GC/CC vs. GG | 366/361 | 1.05 (0.84-1.31) | 1.03 (0.82-1.29) | 0.82 | 541/671 | 0.86 (0.55-1.34) | 0.86 (0.54-1.35) | 0.50 |  |
|  |  |  |  | CC vs. GG/GC | 546/553 | 1.08 (0.78-1.49) | 1.04 (0.75-1.44) | 0.83 | 260/320 | 1.02 (0.82-1.27) | 1.02 (0.81-1.28) | 0.88 |  |
| ALOX5AP | rs4769872 | 13 | 31312630 | GG | 550/532 | 1.00 (ref) | 1.00 (ref) | 0.08 | 351/430 | 1.00 (ref) | 1.00 (ref) | 0.69 | 0.31 |
|  |  |  |  | GA | 83/92 | 0.86 (0.62-1.19) | 0.82 (0.59-1.14) |  | 203/237 | 1.02 (0.81-1.3) | 1.02 (0.80-1.29) |  |  |
|  |  |  |  | AA | 3/10 | 0.30 (0.08-1.09) | 0.27 (0.07-1.01) |  | 29/46 | 0.81 (0.5-1.32) | 0.81 (0.50-1.33) |  |  |
|  |  |  |  | Per copy variant allele |  | 0.78 (0.59-1.04) | 0.74 (0.55-0.99) | **0.04** |  | 0.96 (0.80-1.16) | 0.96 (0.80-1.15) | 0.66 |  |
|  |  |  |  | GA/AA vs. GG | 86/102 | 0.80 (0.59-1.10) | 0.76 (0.55-1.05) | 0.10 | 232/283 | 0.99 (0.79-1.24) | 0.99 (0.78-1.24) | 0.90 |  |
|  |  |  |  | AA vs. GG/GA | 633/624 | 0.30 (0.08-1.11) | 0.28 (0.07-1.03) | 0.06 | 554/667 | 0.80 (0.50-1.30) | 0.81 (0.50-1.31) | 0.39 |  |
| ALOX5AP | rs9315045 | 13 | 31313889 | TT | 355/356 | 1.00 (ref) | 1.00 (ref) | 0.85 | 184/260 | 1.00 (ref) | 1.00 (ref) | 0.32 | 0.61 |
|  |  |  |  | TC | 230/238 | 0.97 (0.77-1.23) | 0.98 (0.77-1.24) |  | 297/336 | 1.18 (0.92-1.52) | 1.18 (0.92-1.51) |  |  |
|  |  |  |  | CC | 51/43 | 1.18 (0.76-1.82) | 1.12 (0.71-1.74) |  | 102/114 | 1.25 (0.89-1.73) | 1.24 (0.89-1.73) |  |  |
|  |  |  |  | Per copy variant allele |  | 1.03 (0.87-1.23) | 1.02 (0.85-1.22) | 0.84 |  | 1.13 (0.96-1.32) | 1.12 (0.96-1.32) | 0.15 |  |
|  |  |  |  | TC/CC vs. TT | 281/281 | 1.00 (0.80-1.25) | 1.00 (0.79-1.25) | 0.98 | 399/450 | 1.20 (0.95-1.52) | 1.19 (0.94-1.51) | 0.14 |  |
|  |  |  |  | CC vs. TT/TC | 585/594 | 1.19 (0.78-1.82) | 1.13 (0.73-1.74) | 0.59 | 481/596 | 1.13 (0.84-1.52) | 1.13 (0.84-1.51) | 0.44 |  |
| ALOX5AP | rs12431114 | 13 | 31317452 | AA | 550/553 | 1.00 (ref) | 1.00 (ref) | 0.44 | 536/653 | 1.00 (ref) | 1.00 (ref) | 0.95 | 0.84 |
|  |  |  |  | AG | 84/78 | 1.10 (0.79-1.54) | 1.11 (0.79-1.56) |  | 45/60 | 0.95 (0.63-1.43) | 0.96 (0.64-1.44) |  |  |
|  |  |  |  | GG | 3/6 | 0.51 (0.12-2.04) | 0.42 (0.09-1.92) |  | 1/2 | 0.72 (0.06-8.20) | 0.74 (0.07-8.46) |  |  |
|  |  |  |  | Per copy variant allele |  | 1.01 (0.75-1.37) | 1.01 (0.74-1.38) | 0.95 |  | 0.94 (0.64-1.38) | 0.95 (0.64-1.40) | 0.78 |  |
|  |  |  |  | AG/GG vs. AA | 87/84 | 1.06 (0.76-1.47) | 1.06 (0.76-1.48) | 0.73 | 46/62 | 0.94 (0.63-1.41) | 0.95 (0.64-1.42) | 0.81 |  |
|  |  |  |  | GG vs. AA/AG | 634/631 | 0.50 (0.12-2.02) | 0.42 (0.09-1.89) | 0.26 | 581/713 | 0.72 (0.06-8.24) | 0.74 (0.07-8.49) | 0.81 |  |
| ALOX5AP | rs4360791 | 13 | 31318020 | GG | 181/196 | 1.00 (ref) | 1.00 (ref) | 0.57 | 111/135 | 1.00 (ref) | 1.00 (ref) | 0.98 | 0.72 |
|  |  |  |  | GA | 313/313 | 1.09 (0.85-1.42) | 1.11 (0.85-1.44) |  | 271/328 | 1.01 (0.74-1.36) | 1.01 (0.75-1.37) |  |  |
|  |  |  |  | AA | 142/124 | 1.23 (0.90-1.69) | 1.18 (0.86-1.64) |  | 198/238 | 1.04 (0.75-1.42) | 1.03 (0.75-1.42) |  |  |
|  |  |  |  | Per copy variant allele |  | 1.11 (0.95-1.30) | 1.09 (0.93-1.28) | 0.29 |  | 1.02 (0.87-1.19) | 1.02 (0.87-1.19) | 0.83 |  |
|  |  |  |  | GA/AA vs. GG | 455/437 | 1.13 (0.89-1.44) | 1.13 (0.88-1.45) | 0.34 | 469/566 | 1.02 (0.77-1.35) | 1.02 (0.77-1.35) | 0.89 |  |
|  |  |  |  | AA vs. GG/GA | 494/509 | 1.16 (0.89-1.53) | 1.11 (0.84-1.47) | 0.46 | 382/463 | 1.03 (0.82-1.31) | 1.02 (0.81-1.30) | 0.84 |  |
| ALOX5AP | rs9579648 | 13 | 31327032 | GG | 453/441 | 1.00 (ref) | 1.00 (ref) | 0.65 | 394/473 | 1.00 (ref) | 1.00 (ref) | 0.24 | 0.25 |
|  |  |  |  | GC | 169/177 | 0.92 (0.72-1.18) | 0.92 (0.71-1.19) |  | 168/217 | 0.94 (0.74-1.20) | 0.95 (0.74-1.22) |  |  |
|  |  |  |  | CC | 13/19 | 0.70 (0.34-1.45) | 0.76 (0.36-1.57) |  | 21/16 | 1.73 (0.88-3.38) | 1.73 (0.88-3.39) |  |  |
|  |  |  |  | Per copy variant allele |  | 0.89 (0.72-1.11) | 0.90 (0.73-1.13) | 0.37 |  | 1.05 (0.85-1.29) | 1.06 (0.86-1.30) | 0.59 |  |
|  |  |  |  | GC/CC vs. GG | 182/196 | 0.90 (0.71-1.15) | 0.91 (0.71-1.16) | 0.43 | 189/233 | 0.99 (0.78-1.26) | 1.00 (0.79-1.27) | 0.98 |  |
|  |  |  |  | CC vs. GG/GC | 622/618 | 0.72 (0.35-1.48) | 0.77 (0.37-1.60) | 0.49 | 562/690 | 1.76 (0.90-3.43) | 1.76 (0.90-3.43) | 0.10 |  |
| ALOX5AP | rs9315048 | 13 | 31327840 | GG | 368/388 | 1.00 (ref) | 1.00 (ref) | 0.14 | 270/354 | 1.00 (ref) | 1.00 (ref) | 0.37 | 0.65 |
|  |  |  |  | GT | 220/220 | 1.08 (0.85-1.36) | 1.09 (0.86-1.39) |  | 245/294 | 1.09 (0.86-1.38) | 1.08 (0.86-1.37) |  |  |
|  |  |  |  | TT | 49/29 | 1.70 (1.05-2.76) | 1.64 (1.00-2.69) |  | 63/63 | 1.32 (0.90-1.94) | 1.31 (0.89-1.94) |  |  |
|  |  |  |  | Per copy variant allele |  | 1.18 (0.99-1.42) | 1.18 (0.98-1.42) | 0.08 |  | 1.13 (0.95-1.33) | 1.12 (0.95-1.33) | 0.18 |  |
|  |  |  |  | GT/TT vs. GG | 269/249 | 1.15 (0.92-1.44) | 1.16 (0.92-1.46) | 0.21 | 308/357 | 1.13 (0.90-1.41) | 1.12 (0.90-1.40) | 0.31 |  |
|  |  |  |  | TT vs. GG/GT | 588/608 | 1.66 (1.03-2.67) | 1.58 (0.97-2.58) | 0.06 | 515/648 | 1.27 (0.87-1.84) | 1.27 (0.87-1.84) | 0.22 |  |
| ALOX5AP | rs9741777 | 13 | 31337750 | AA | 596/607 | 1.00 (ref) | 1.00 (ref) | 0.51 | 551/665 | 1.00 (ref) | 1.00 (ref) | 0.54 | 0.27 |
|  |  |  |  | AG | 40/29 | 1.37 (0.83-2.25) | 1.32 (0.80-2.20) |  | 33/49 | 0.76 (0.48-1.21) | 0.77 (0.49-1.22) |  |  |
|  |  |  |  | GG | 1/2 | 0.52 (0.05-5.85) | 0.62 (0.05-7.06) |  | 0/1 |  |  |  |  |
|  |  |  |  | Per copy variant allele |  | 1.24 (0.79-1.96) | 1.22 (0.77-1.94) | 0.40 |  | 0.74 (0.47-1.17) | 0.75 (0.47-1.18) | 0.21 |  |
|  |  |  |  | AG/GG vs. AA | 41/31 | 1.31 (0.81-2.14) | 1.28 (0.78-2.11) | 0.33 | 33/50 | 0.75 (0.47-1.19) | 0.76 (0.48-1.20) | 0.24 |  |
|  |  |  |  | GG vs. AA/AG | 636/636 | 0.51 (0.05-5.76) | 0.61 (0.05-6.96) | 0.69 |  |  |  |  |  |
| ALOX5AP | rs1132340 | 13 | 31338462 | AA | 578/560 | 1.00 (ref) | 1.00 (ref) | 0.14 | 431/522 | 1.00 (ref) | 1.00 (ref) | 0.21 | 0.35 |
|  |  |  |  | AG | 56/74 | 0.72 (0.50-1.04) | 0.68 (0.46-0.99) |  | 141/170 | 1.00 (0.77-1.29) | 1.00 (0.77-1.30) |  |  |
|  |  |  |  | GG | 0/3 |  |  |  | 9/21 | 0.49 (0.22-1.09) | 0.49 (0.22-1.09) |  |  |
|  |  |  |  | Per copy variant allele |  | 0.68 (0.47-0.97) | 0.65 (0.45-0.93) | **0.02** |  | 0.90 (0.72-1.12) | 0.90 (0.72-1.12) | 0.35 |  |
|  |  |  |  | AG/GG vs. AA | 56/77 | 0.69 (0.48-1.00) | 0.66 (0.45-0.96) | **0.03** | 150/191 | 0.94 (0.73-1.21) | 0.94 (0.73-1.21) | 0.65 |  |
|  |  |  |  | GG vs. AA/AG |  |  |  |  | 572/692 | 0.49 (0.22-1.09) | 0.49 (0.22-1.08) | 0.08 |  |
| ALOX12 | rs3840880^h^ | 17 | 6897844 | TT | 221/187 | 1.00 (ref) | 1.00 (ref) | 0.08 | 133/159 | 1.00 (ref) | 1.00 (ref) | 0.94 | 0.19 |
|  |  |  |  | TG | 313/316 | 0.84 (0.65-1.08) | 0.88 (0.68-1.13) |  | 279/345 | 0.97 (0.73-1.28) | 0.96 (0.73-1.28) |  |  |
|  |  |  |  | GG | 101/131 | 0.64 (0.46-0.89) | 0.68 (0.49-0.95) |  | 171/211 | 1.00 (0.73-1.36) | 1.01 (0.74-1.38) |  |  |
|  |  |  |  | Per copy variant allele |  | 0.81 (0.69-0.95) | 0.83 (0.71-0.98) | **0.03** |  | 1.00 (0.86-1.17) | 1.01 (0.86-1.18) | 0.94 |  |
|  |  |  |  | TG/GG vs. TT | 414/447 | 0.78 (0.62-0.99) | 0.82 (0.64-1.04) | 0.11 | 450/556 | 0.98 (0.75-1.28) | 0.98 (0.75-1.28) | 0.88 |  |
|  |  |  |  | GG vs. TT/TG | 534/503 | 0.72 (0.54-0.96) | 0.74 (0.55-0.99) | **0.04** | 412/504 | 1.02 (0.80-1.31) | 1.03 (0.81-1.32) | 0.80 |  |
| ALOX12 | rs2292350 | 17 | 6901672 | GG | 198/207 | 1.00 (ref) | 1.00 (ref) | 0.69 | 462/542 | 1.00 (ref) | 1.00 (ref) | 0.20 | 0.39 |
|  |  |  |  | GA | 313/318 | 1.05 (0.81-1.35) | 1.06 (0.82-1.36) |  | 112/159 | 0.81 (0.61-1.06) | 0.80 (0.60-1.06) |  |  |
|  |  |  |  | AA | 124/110 | 1.19 (0.86-1.65) | 1.16 (0.83-1.61) |  | 8/14 | 0.66 (0.27-1.60) | 0.64 (0.26-1.56) |  |  |
|  |  |  |  | Per copy variant allele |  | 1.08 (0.92-1.27) | 1.07 (0.91-1.26) | 0.40 |  | 0.81 (0.64-1.03) | 0.80 (0.62-1.02) | 0.07 |  |
|  |  |  |  | GA/AA vs. GG | 437/428 | 1.08 (0.85-1.37) | 1.08 (0.85-1.38) | 0.52 | 120/173 | 0.80 (0.61-1.04) | 0.78 (0.60-1.03) | 0.08 |  |
|  |  |  |  | AA vs. GG/GA | 511/525 | 1.16 (0.87-1.54) | 1.12 (0.84-1.50) | 0.45 | 574/701 | 0.69 (0.29-1.67) | 0.68 (0.28-1.67) | 0.40 |  |
| ALOX12 | rs1126667^h^ | 17 | 6902760 | GG | 227/191 | 1.00 (ref) | 1.00 (ref) | 0.12 | 264/298 | 1.00 (ref) | 1.00 (ref) | 0.31 | 0.27 |
|  |  |  |  | GA | 310/318 | 0.81 (0.63-1.04) | 0.86 (0.66-1.11) |  | 247/328 | 0.84 (0.66-1.06) | 0.83 (0.66-1.06) |  |  |
|  |  |  |  | AA | 99/126 | 0.64 (0.46-0.90) | 0.71 (0.51-0.99) |  | 71/86 | 0.94 (0.66-1.35) | 0.94 (0.66-1.35) |  |  |
|  |  |  |  | Per copy variant allele |  | 0.80 (0.68-0.94) | 0.84 (0.72-0.99) | **0.04** |  | 0.93 (0.79-1.09) | 0.93 (0.79-1.09) | 0.36 |  |
|  |  |  |  | GA/AA vs. GG | 409/444 | 0.76 (0.60-0.97) | 0.82 (0.64-1.04) | 0.10 | 318/414 | 0.86 (0.69-1.07) | 0.86 (0.68-1.07) | 0.17 |  |
|  |  |  |  | AA vs. GG/GA | 537/509 | 0.73 (0.54-0.98) | 0.78 (0.58-1.04) | 0.09 | 511/626 | 1.03 (0.73-1.44) | 1.03 (0.74-1.45) | 0.84 |  |
| ALOX12 | rs434473^h^ | 17 | 6904934 | AA | 230/190 | 1.00 (ref) | 1.00 (ref) | 0.09 | 393/474 | 1.00 (ref) | 1.00 (ref) | 0.94 | 0.21 |
|  |  |  |  | AG | 308/319 | 0.78 (0.61-1.01) | 0.85 (0.65-1.09) |  | 163/204 | 0.96 (0.75-1.23) | 0.96 (0.75-1.23) |  |  |
|  |  |  |  | GG | 97/125 | 0.62 (0.45-0.86) | 0.69 (0.49-0.96) |  | 28/35 | 1.01 (0.60-1.70) | 1.01 (0.60-1.70) |  |  |
|  |  |  |  | Per copy variant allele |  | 0.79 (0.67-0.92) | 0.83 (0.71-0.98) | **0.03** |  | 0.98 (0.81-1.19) | 0.98 (0.81-1.19) | 0.83 |  |
|  |  |  |  | AG/GG vs. AA | 405/444 | 0.74 (0.58-0.94) | 0.80 (0.63-1.02) | 0.07 | 191/239 | 0.97 (0.77-1.23) | 0.96 (0.76-1.22) | 0.76 |  |
|  |  |  |  | GG vs. AA/AG | 538/509 | 0.72 (0.53-0.96) | 0.76 (0.57-1.03) | 0.08 | 556/678 | 1.02 (0.61-1.71) | 1.02 (0.61-1.72) | 0.93 |  |
| ALOX12 | rs1042357^h^ | 17 | 6905061 | GG | 219/184 | 1.00 (ref) | 1.00 (ref) | 0.10 | 204/223 | 1.00 (ref) | 1.00 (ref) | 0.27 | 0.65 |
|  |  |  |  | GT | 314/326 | 0.80 (0.62-1.03) | 0.85 (0.66-1.11) |  | 276/350 | 0.83 (0.65-1.07) | 0.83 (0.65-1.07) |  |  |
|  |  |  |  | TT | 100/127 | 0.64 (0.46-0.90) | 0.70 (0.50-0.97) |  | 102/139 | 0.81 (0.59-1.12) | 0.81 (0.59-1.12) |  |  |
|  |  |  |  | Per copy variant allele |  | 0.80 (0.68-0.94) | 0.84 (0.71-0.99) | **0.03** |  | 0.89 (0.76-1.04) | 0.89 (0.76-1.04) | 0.14 |  |
|  |  |  |  | GT/TT vs. GG | 414/453 | 0.76 (0.60-0.96) | 0.81 (0.63-1.03) | 0.09 | 378/489 | 0.83 (0.65-1.05) | 0.82 (0.65-1.04) | 0.11 |  |
|  |  |  |  | TT vs. GG/GT | 533/510 | 0.74 (0.55-0.99) | 0.77 (0.57-1.03) | 0.08 | 480/573 | 0.91 (0.68-1.21) | 0.90 (0.68-1.20) | 0.49 |  |
| ALOX12 | rs312462 | 17 | 6913652 | CC | 518/493 | 1.00 (ref) | 1.00 (ref) | 0.32 | 518/627 | 1.00 (ref) | 1.00 (ref) | 0.54 | 0.33 |
|  |  |  |  | CT | 112/135 | 0.77 (0.58-1.02) | 0.81 (0.61-1.08) |  | 62/86 | 0.91 (0.64-1.30) | 0.91 (0.64-1.30) |  |  |
|  |  |  |  | TT | 7/10 | 0.73 (0.27-1.95) | 0.80 (0.30-2.14) |  | 4/2 | 2.33 (0.42-13.07) | 2.35 (0.41-13.36) |  |  |
|  |  |  |  | Per copy variant allele |  | 0.79 (0.61-1.01) | 0.83 (0.64-1.07) | 0.14 |  | 0.98 (0.71-1.36) | 0.98 (0.71-1.36) | 0.92 |  |
|  |  |  |  | CT/TT vs. CC | 119/145 | 0.76 (0.58-1.01) | 0.81 (0.61-1.07) | 0.13 | 66/88 | 0.95 (0.67-1.33) | 0.95 (0.67-1.33) | 0.75 |  |
|  |  |  |  | TT vs. CC/CT | 630/628 | 0.77 (0.29-2.05) | 0.83 (0.31-2.23) | 0.71 | 580/713 | 2.36 (0.42-13.20) | 2.38 (0.42-13.52) | 0.33 |  |

^a^ OR, odds ratio; 95%CI, 95% confidence interval

^b^ Adjusted for age
^c^ Adjusted for age, family history of breast cancer in a first-degree relative, and proportion of European ancestry

^d^ *P*-trend for genetic dose response determined by coding genotypes as having 0, 1, or 2 variant allele, which was subsequently analyzed as an ordinal variable

^e^ *P* for heterogeneity from dominant or recessive models

^f^ All significant p-values were further adjusted for multiple comparisons using Bonferroni correction, with *P*<0.002 (0.05/30) considered statistically significant.

^g^ *P* for interaction term including genotype and race in the multivariable logistic model ^h^ Several SNPs on the ALOX12 gene, rs3840880, rs1126667, rs434473, rs1042357, were found in high LD with rs3840880 (r^2^>0.98) in white women, with a similar association pattern

**Supplemental Table S2. SNPs of inflammation related pathways and risk of breast cancer by menopausal status** **in the WCHS**

| **Gene** | **SNP** | **Genotype** | **White** | | | | | | **Black** | | | | | |
| --- | --- | --- | --- | --- | --- | --- | --- | --- | --- | --- | --- | --- | --- | --- |
|  |  |  | **Pre-menopausal women** | | | **Post-menopausal women** | | | **Pre-menopausal women** | | | **Post-menopausal women** | | |
|  |  |  | **#Case/**  **Control** | **OR**  **(95% CI)^a,b^** | **P^c,d,e^** | **#Case/**  **Control** | **OR**  **(95% CI)^a,b^** | **P^c,d,e^** | **#Case/**  **Control** | **OR**  **(95% CI)^a,b^** | **P^c,d,e^** | **#Case/**  **Control** | **OR**  **(95% CI)^a,b^** | **P^c,d,e^** |
| COX2 | rs689470 | CC | 310/338 | 1.00 (ref) | 0.07 | 279/280 | 1.00 (ref) | **0.02** | 103/158 | 1.00 (ref) | 0.49 | 102/104 | 1.00 (ref) | 0.99 |
|  |  | CT | 20/9 | 2.01 (0.86-4.69) |  | 27/8 | 2.72 (1.16-6.40) |  | 124/160 | 1.23 (0.87-1.74) |  | 149/165 | 1.00 (0.69-1.44) |  |
|  |  | TT | 1/3 | 0.03 (0.00-2.24) |  | 0/0 |  |  | 56/74 | 1.19 (0.77-1.84) |  | 46/53 | 0.96 (0.58-1.60) |  |
|  |  | Per copy variant allele |  | 1.10 (0.56-2.17) | 0.78 |  | 2.72 (1.16-6.40) | **0.02** |  | 1.11 (0.90-1.37) | 0.33 |  | 0.98 (0.77-1.26) | 0.90 |
|  |  | CT/TT vs. CC | 21/12 | 1.46 (0.67-3.19) | 0.34 | 27/8 | 2.72 (1.16-6.40) | **0.02** | 180/234 | 1.22 (0.88-1.68) | 0.23 | 195/218 | 0.99 (0.69-1.41) | 0.95 |
|  |  | TT vs. CC/CT | 330/347 | 0.03 (0.00-2.25) | 0.11 |  |  |  | 227/318 | 1.07 (0.72-1.58) | 0.75 | 251/269 | 0.96 (0.62-1.51) | 0.88 |
| COX2 | rs2206593 | GG | 288/298 | 1.00 (ref) | 0.76 | 262/244 | 1.00 (ref) | 0.91 | 282/384 | 1.00 (ref) | 0.32 | 295/317 | 1.00 (ref) | 0.46 |
|  |  | GA | 38/48 | 0.84 (0.53-1.34) |  | 40/41 | 0.90 (0.55-1.47) |  | 3/8 | 0.51 (0.13-1.96) |  | 2/5 | 0.53 (0.10-2.81) |  |
|  |  | AA | 3/4 | 0.82 (0.18-3.75) |  | 3/0 |  |  | 0/0 |  |  | 0/0 |  |  |
|  |  | Per copy variant allele |  | 0.86 (0.57-1.29) | 0.47 |  | 1.05 (0.66-1.66) | 0.84 |  | 0.51 (0.13-1.96) | 0.32 |  | 0.53 (0.10-2.81) | 0.46 |
|  |  | GA/AA vs. GG | 41/52 | 0.84 (0.54-1.32) | 0.45 | 43/41 | 0.97 (0.60-1.58) | 0.90 | 3/8 | 0.51 (0.13-1.96) | 0.32 | 2/5 | 0.53 (0.10-2.81) | 0.46 |
|  |  | AA vs. GG/GA | 326/346 | 0.84 (0.19-3.83) | 0.82 |  |  |  |  |  |  |  |  |  |
| COX2 | rs5275 | TT | 149/162 | 1.00 (ref) | 0.65 | 120/117 | 1.00 (ref) | 0.29 | 54/65 | 1.00 (ref) | 0.75 | 38/54 | 1.00 (ref) | 0.10 |
|  |  | TC | 140/155 | 0.97 (0.70-1.35) |  | 153/133 | 1.17 (0.81-1.68) |  | 124/174 | 0.88 (0.57-1.36) |  | 147/163 | 1.24 (0.76-2.02) |  |
|  |  | CC | 40/33 | 1.25 (0.74-2.12) |  | 31/37 | 0.75 (0.42-1.34) |  | 103/152 | 0.84 (0.54-1.31) |  | 110/99 | 1.69 (1.01-2.84) |  |
|  |  | Per copy variant allele |  | 1.06 (0.84-1.34) | 0.60 |  | 0.96 (0.74-1.24) | 0.73 |  | 0.92 (0.74-1.15) | 0.47 |  | 1.31 (1.02-1.69) | **0.03** |
|  |  | TC/CC vs. TT | 180/188 | 1.02 (0.75-1.39) | 0.90 | 184/170 | 1.07 (0.76-1.52) | 0.68 | 227/326 | 0.86 (0.58-1.29) | 0.48 | 257/262 | 1.40 (0.88-2.23) | 0.16 |
|  |  | CC vs. TT/TC | 289/317 | 1.27 (0.76-2.09) | 0.36 | 273/250 | 0.69 (0.40-1.19) | 0.18 | 178/239 | 0.92 (0.67-1.27) | 0.61 | 185/217 | 1.43 (1-2.03) | **0.047** |
| COX2 | rs4648274 | AA | 330/347 | 1.00 (ref) | 0.054 | 301/286 | 1.00 (ref) | 0.59 | 213/299 | 1.00 (ref) | 0.89 | 226/242 | 1.00 (ref) | 0.78 |
|  |  | AC | 1/3 | 0.01 (0.00-1.07) |  | 3/1 | 1.99 (0.16-24.22) |  | 62/83 | 1.09 (0.74-1.58) |  | 67/77 | 1.00 (0.68-1.48) |  |
|  |  | CC | 0/0 |  |  | 0/0 |  |  | 8/10 | 1.14 (0.44-2.98) |  | 4/2 | 1.87 (0.32-10.8) |  |
|  |  | Per copy variant allele |  | 0.01 (0.00-1.07) | 0.054 |  | 1.99 (0.16-24.22) | 0.59 |  | 1.08 (0.79-1.47) | 0.63 |  | 1.05 (0.73-1.51) | 0.79 |
|  |  | AC/CC vs. AA | 1/3 | 0.01 (0.00-1.07) | 0.054 | 3/1 | 1.99 (0.16-24.22) | 0.59 | 70/93 | 1.09 (0.76-1.57) | 0.63 | 71/79 | 1.02 (0.70-1.50) | 0.90 |
|  |  | CC vs. AA/AC |  |  |  |  |  |  | 275/382 | 1.12 (0.43-2.91) | 0.81 | 293/319 | 1.87 (0.32-10.8) | 0.48 |
| COX2 | rs2745557 | GG | 214/223 | 1.00 (ref) | 0.27 | 190/174 | 1.00 (ref) | 0.84 | 200/284 | 1.00 (ref) | 0.69 | 230/219 | 1.00 (ref) | **0.03** |
|  |  | GA | 97/101 | 1.07 (0.76-1.52) |  | 100/102 | 0.94 (0.65-1.35) |  | 76/94 | 1.14 (0.80-1.63) |  | 61/96 | 0.60 (0.41-0.88) |  |
|  |  | AA | 18/26 | 0.61 (0.31-1.18) |  | 16/11 | 1.20 (0.52-2.78) |  | 9/15 | 0.85 (0.36-1.99) |  | 7/6 | 1.16 (0.38-3.58) |  |
|  |  | Per copy variant allele |  | 0.90 (0.70-1.17) | 0.44 |  | 1.00 (0.75-1.34) | 0.99 |  | 1.05 (0.79-1.39) | 0.75 |  | 0.71 (0.51-0.99) | **0.045** |
|  |  | GA/AA vs. GG | 115/127 | 0.97 (0.70-1.34) | 0.85 | 116/113 | 0.97 (0.68-1.37) | 0.85 | 85/109 | 1.10 (0.79-1.55) | 0.58 | 68/102 | 0.63 (0.44-0.92) | **0.02** |
|  |  | AA vs. GG/GA | 311/324 | 0.59 (0.31-1.14) | 0.12 | 290/276 | 1.23 (0.53-2.81) | 0.63 | 276/378 | 0.82 (0.35-1.92) | 0.65 | 291/315 | 1.32 (0.43-4.06) | 0.63 |
| COX2 | rs689466 | AA | 216/241 | 1.00 (ref) | 0.59 | 212/210 | 1.00 (ref) | 0.47 | 237/323 | 1.00 (ref) | 0.88 | 242/259 | 1.00 (ref) | 0.91 |
|  |  | AG | 101/100 | 1.15 (0.82-1.62) |  | 82/69 | 1.19 (0.80-1.77) |  | 45/65 | 0.93 (0.61-1.42) |  | 51/59 | 0.91 (0.60-1.40) |  |
|  |  | GG | 11/9 | 1.41 (0.56-3.58) |  | 11/7 | 1.63 (0.60-4.44) |  | 2/4 | 0.71 (0.13-3.98) |  | 4/3 | 1.10 (0.24-5.09) |  |
|  |  | Per copy variant allele |  | 1.16 (0.87-1.55) | 0.31 |  | 1.22 (0.88-1.69) | 0.22 |  | 0.92 (0.63-1.34) | 0.65 |  | 0.94 (0.64-1.38) | 0.76 |
|  |  | AG/GG vs. AA | 112/109 | 1.17 (0.84-1.63) | 0.35 | 93/76 | 1.23 (0.84-1.80) | 0.28 | 47/69 | 0.92 (0.61-1.39) | 0.70 | 55/62 | 0.92 (0.61-1.4) | 0.71 |
|  |  | GG vs. AA/AG | 317/341 | 1.35 (0.54-3.41) | 0.52 | 294/279 | 1.56 (0.58-4.21) | 0.38 | 282/388 | 0.72 (0.13-4.02) | 0.71 | 293/318 | 1.12 (0.24-5.17) | 0.88 |
| ALOX5 | rs6593482 | GG | 233/247 | 1.00 (ref) | 0.75 | 204/207 | 1.00 (ref) | 0.20 | 190/278 | 1.00 (ref) | 0.63 | 206/214 | 1.00 (ref) | 0.89 |
|  |  | GT | 79/87 | 1.03 (0.72-1.48) |  | 93/77 | 1.36 (0.93-2.00) |  | 83/97 | 1.18 (0.83-1.68) |  | 81/94 | 0.94 (0.65-1.35) |  |
|  |  | TT | 16/12 | 1.36 (0.61-3.02) |  | 6/3 | 1.97 (0.46-8.41) |  | 9/13 | 0.96 (0.40-2.31) |  | 9/10 | 0.84 (0.33-2.18) |  |
|  |  | Per copy variant allele |  | 1.09 (0.82-1.45) | 0.55 |  | 1.37 (0.97-1.94) | 0.07 |  | 1.10 (0.83-1.47) | 0.51 |  | 0.93 (0.69-1.26) | 0.64 |
|  |  | GT/TT vs. GG | 95/99 | 1.07 (0.76-1.51) | 0.69 | 99/80 | 1.39 (0.96-2.02) | 0.09 | 92/110 | 1.16 (0.83-1.62) | 0.39 | 90/104 | 0.93 (0.65-1.32) | 0.67 |
|  |  | TT vs. GG/GT | 312/334 | 1.35 (0.61-2.98) | 0.46 | 297/284 | 1.80 (0.42-7.64) | 0.43 | 273/375 | 0.92 (0.38-2.19) | 0.85 | 287/308 | 0.86 (0.34-2.21) | 0.75 |
| ALOX5 | rs7099684 | TT | 203/216 | 1.00 (ref) | 0.82 | 193/169 | 1.00 (ref) | 0.36 | 236/309 | 1.00 (ref) | 0.26 | 234/243 | 1.00 (ref) | 0.64 |
|  |  | TA | 113/122 | 1.00 (0.72-1.39) |  | 99/107 | 0.76 (0.53-1.10) |  | 47/75 | 0.84 (0.56-1.26) |  | 59/75 | 0.85 (0.57-1.26) |  |
|  |  | AA | 15/12 | 1.30 (0.58-2.92) |  | 13/12 | 0.90 (0.39-2.11) |  | 2/9 | 0.31 (0.07-1.48) |  | 4/3 | 1.40 (0.3-6.53) |  |
|  |  | Per copy variant allele |  | 1.05 (0.80-1.38) | 0.72 |  | 0.84 (0.62-1.13) | 0.24 |  | 0.76 (0.54-1.09) | 0.14 |  | 0.90 (0.63-1.3) | 0.59 |
|  |  | TA/AA vs. TT | 128/134 | 1.03 (0.75-1.41) | 0.87 | 112/119 | 0.78 (0.55-1.11) | 0.17 | 49/84 | 0.79 (0.53-1.17) | 0.23 | 63/78 | 0.87 (0.59-1.28) | 0.48 |
|  |  | AA vs. TT/TA | 316/338 | 1.30 (0.58-2.89) | 0.53 | 292/276 | 0.99 (0.43-2.28) | 0.98 | 283/384 | 0.32 (0.07-1.53) | 0.15 | 293/318 | 1.46 (0.31-6.77) | 0.63 |
| ALOX5 | rs1369214 | GG | 100/101 | 1.00 (ref) | 0.94 | 95/89 | 1.00 (ref) | 0.61 | 42/87 | 1.00 (ref) | **0.02** | 70/69 | 1.00 (ref) | 0.18 |
|  |  | GA | 158/172 | 0.96 (0.67-1.38) |  | 158/138 | 1.18 (0.80-1.75) |  | 140/197 | 1.39 (0.90-2.14) |  | 134/170 | 0.81 (0.53-1.22) |  |
|  |  | AA | 72/76 | 0.92 (0.59-1.43) |  | 51/58 | 0.99 (0.59-1.63) |  | 102/108 | 1.90 (1.20-3.01) |  | 94/81 | 1.15 (0.72-1.82) |  |
|  |  | Per copy variant allele |  | 0.96 (0.77-1.20) | 0.71 |  | 1.02 (0.79-1.30) | 0.90 |  | 1.38 (1.10-1.72) | **0.005** |  | 1.09 (0.87-1.37) | 0.46 |
|  |  | GA/AA vs. GG | 230/248 | 0.95 (0.67-1.33) | 0.75 | 209/196 | 1.13 (0.78-1.63) | 0.53 | 242/305 | 1.57 (1.05-2.37) | **0.03** | 228/251 | 0.92 (0.62-1.36) | 0.67 |
|  |  | AA vs. GG/GA | 258/273 | 0.95 (0.65-1.38) | 0.78 | 253/227 | 0.89 (0.57-1.38) | 0.60 | 182/284 | 1.50 (1.07-2.09) | **0.02** | 204/239 | 1.33 (0.93-1.91) | 0.12 |
| ALOX5 | rs2228065 | GG | 328/348 | 1.00 (ref) | 0.32 | 304/288 | 1.00 (ref) | 0.99 | 251/339 | 1.00 (ref) | 0.42 | 243/266 | 1.00 (ref) | 0.99 |
|  |  | GA | 2/2 | 0.22 (0.01-4.15) |  | 1/0 |  |  | 33/48 | 0.96 (0.60-1.55) |  | 52/56 | 1.03 (0.67-1.58) |  |
|  |  | AA | 0/0 |  |  | 0/0 |  |  | 1/6 | 0.24 (0.03-2.04) |  | 3/0 |  |  |
|  |  | Per copy variant allele |  | 0.22 (0.01-4.15) | 0.32 |  |  |  |  | 0.83 (0.55-1.27) | 0.39 |  | 1.17 (0.78-1.76) | 0.45 |
|  |  | GA/AA vs. GG | 2/2 | 0.22 (0.01-4.15) | 0.32 |  |  |  | 34/54 | 0.88 (0.55-1.40) | 0.60 | 55/56 | 1.10 (0.72-1.68) | 0.67 |
|  |  | AA vs. GG/GA |  |  |  |  |  |  | 284/387 | 0.24 (0.03-2.05) | 0.19 |  |  |  |
| ALOX5 | rs1487562 | CC | 218/241 | 1.00 (ref) | 0.30 | 201/187 | 1.00 (ref) | 0.40 | 154/222 | 1.00 (ref) | 0.78 | 183/178 | 1.00 (ref) | **0.049** |
|  |  | CT | 91/98 | 1.13 (0.80-1.60) |  | 89/92 | 0.94 (0.65-1.37) |  | 110/140 | 1.12 (0.81-1.55) |  | 101/118 | 0.80 (0.56-1.13) |  |
|  |  | TT | 21/11 | 1.79 (0.82-3.92) |  | 16/9 | 1.76 (0.73-4.28) |  | 21/31 | 0.97 (0.53-1.76) |  | 13/26 | 0.43 (0.21-0.88) |  |
|  |  | Per copy variant allele |  | 1.22 (0.93-1.60) | 0.16 |  | 1.08 (0.80-1.46) | 0.60 |  | 1.04 (0.82-1.32) | 0.75 |  | 0.72 (0.55-0.95) | **0.02** |
|  |  | CT/TT vs. CC | 112/109 | 1.20 (0.86-1.67) | 0.27 | 105/101 | 1.01 (0.71-1.45) | 0.95 | 131/171 | 1.09 (0.80-1.48) | 0.59 | 114/144 | 0.73 (0.52-1.02) | 0.061 |
|  |  | TT vs. CC/CT | 309/339 | 1.73 (0.80-3.75) | 0.17 | 290/279 | 1.80 (0.75-4.33) | 0.19 | 264/362 | 0.93 (0.52-1.66) | 0.80 | 284/296 | 0.47 (0.23-0.95) | **0.04** |
| ALOX5 | rs7099874 | GG | 167/161 | 1.00 (ref) | 0.52 | 161/120 | 1.00 (ref) | **0.006** | 219/293 | 1.00 (ref) | 0.76 | 210/232 | 1.00 (ref) | 0.71 |
|  |  | GC | 139/157 | 0.87 (0.63-1.21) |  | 110/147 | 0.60 (0.42-0.86) |  | 58/88 | 0.87 (0.60-1.27) |  | 75/77 | 1.17 (0.8-1.72) |  |
|  |  | CC | 22/30 | 0.74 (0.40-1.36) |  | 33/18 | 1.29 (0.67-2.47) |  | 6/9 | 0.95 (0.32-2.79) |  | 11/10 | 1.06 (0.43-2.61) |  |
|  |  | Per copy variant allele |  | 0.87 (0.68-1.11) | 0.26 |  | 0.87 (0.67-1.13) | 0.30 |  | 0.90 (0.65-1.24) | 0.52 |  | 1.11 (0.82-1.51) | 0.50 |
|  |  | GC/CC vs. GG | 161/187 | 0.85 (0.62-1.16) | 0.31 | 143/165 | 0.68 (0.48-0.96) | **0.03** | 64/97 | 0.88 (0.61-1.26) | 0.47 | 86/87 | 1.16 (0.81-1.67) | 0.43 |
|  |  | CC vs. GG/GC | 306/318 | 0.79 (0.44-1.42) | 0.43 | 271/267 | 1.64 (0.88-3.08) | 0.12 | 277/381 | 0.98 (0.33-2.88) | 0.97 | 285/309 | 1.02 (0.41-2.5) | 0.97 |
| ALOX5 | rs1051713 | CC | 227/251 | 1.00 (ref) | 0.56 | 210/189 | 1.00 (ref) | 0.32 | 204/276 | 1.00 (ref) | 0.11 | 189/210 | 1.00 (ref) | 0.58 |
|  |  | CT | 90/89 | 1.18 (0.83-1.69) |  | 83/91 | 0.84 (0.58-1.22) |  | 76/97 | 1.07 (0.75-1.53) |  | 98/96 | 1.17 (0.82-1.66) |  |
|  |  | TT | 11/9 | 1.36 (0.54-3.41) |  | 13/7 | 1.75 (0.64-4.75) |  | 5/19 | 0.36 (0.13-0.97) |  | 10/15 | 0.81 (0.34-1.9) |  |
|  |  | Per copy variant allele |  | 1.18 (0.88-1.58) | 0.28 |  | 0.99 (0.72-1.35) | 0.93 |  | 0.87 (0.65-1.16) | 0.34 |  | 1.05 (0.79-1.4) | 0.73 |
|  |  | CT/TT vs. CC | 101/98 | 1.20 (0.85-1.69) | 0.30 | 96/98 | 0.90 (0.63-1.30) | 0.58 | 81/116 | 0.96 (0.68-1.34) | 0.79 | 108/111 | 1.12 (0.8-1.58) | 0.51 |
|  |  | TT vs. CC/CT | 317/340 | 1.30 (0.52-3.23) | 0.57 | 293/280 | 1.84 (0.68-4.98) | 0.23 | 280/373 | 0.35 (0.13-0.95) | **0.04** | 287/306 | 0.77 (0.33-1.78) | 0.54 |
| ALOX5AP | rs4076128 | AA | 164/171 | 1.00 (ref) | 0.68 | 161/146 | 1.00 (ref) | 0.47 | 20/27 | 1.00 (ref) | 0.51 | 18/17 | 1.00 (ref) | 0.31 |
|  |  | AG | 138/142 | 0.99 (0.71-1.37) |  | 115/120 | 0.82 (0.57-1.17) |  | 92/145 | 0.82 (0.43-1.56) |  | 115/105 | 1.23 (0.58-2.61) |  |
|  |  | GG | 28/34 | 0.78 (0.44-1.38) |  | 28/21 | 1.09 (0.57-2.09) |  | 172/220 | 1.00 (0.53-1.87) |  | 163/197 | 0.93 (0.44-1.97) |  |
|  |  | Per copy variant allele |  | 0.92 (0.73-1.18) | 0.52 |  | 0.94 (0.72-1.23) | 0.65 |  | 1.09 (0.85-1.41) | 0.50 |  | 0.85 (0.64-1.13) | 0.26 |
|  |  | AG/GG vs. AA | 166/176 | 0.95 (0.70-1.30) | 0.74 | 143/141 | 0.86 (0.61-1.21) | 0.38 | 264/365 | 0.92 (0.50-1.69) | 0.78 | 278/302 | 1.06 (0.51-2.21) | 0.87 |
|  |  | GG vs. AA/AG | 302/313 | 0.78 (0.45-1.36) | 0.38 | 276/266 | 1.19 (0.63-2.24) | 0.58 | 112/172 | 1.18 (0.85-1.62) | 0.32 | 133/122 | 0.78 (0.55-1.10) | 0.15 |
| ALOX5AP | rs4073259 | AA | 130/129 | 1.00 (ref) | 0.65 | 127/118 | 1.00 (ref) | 0.59 | 10/20 | 1.00 (ref) | 0.52 | 14/9 | 1.00 (ref) | 0.82 |
|  |  | AG | 149/163 | 0.87 (0.62-1.22) |  | 130/130 | 0.85 (0.58-1.23) |  | 78/119 | 1.20 (0.53-2.72) |  | 98/103 | 0.79 (0.31-1.99) |  |
|  |  | GG | 52/58 | 0.84 (0.53-1.34) |  | 48/39 | 1.05 (0.62-1.77) |  | 195/252 | 1.41 (0.63-3.14) |  | 185/210 | 0.75 (0.30-1.88) |  |
|  |  | Per copy variant allele |  | 0.91 (0.73-1.13) | 0.39 |  | 0.98 (0.77-1.25) | 0.85 |  | 1.18 (0.89-1.57) | 0.25 |  | 0.92 (0.68-1.25) | 0.60 |
|  |  | AG/GG vs. AA | 201/221 | 0.86 (0.63-1.18) | 0.36 | 178/169 | 0.89 (0.63-1.26) | 0.52 | 273/371 | 1.33 (0.60-2.93) | 0.48 | 283/313 | 0.77 (0.31-1.90) | 0.57 |
|  |  | GG vs. AA/AG | 279/292 | 0.91 (0.60-1.39) | 0.67 | 257/248 | 1.14 (0.70-1.86) | 0.59 | 88/139 | 1.20 (0.86-1.68) | 0.28 | 112/112 | 0.93 (0.66-1.33) | 0.70 |
| ALOX5AP | rs4293222 | GG | 136/148 | 1.00 (ref) | 0.89 | 135/129 | 1.00 (ref) | 0.74 | 13/31 | 1.00 (ref) | 0.27 | 28/13 | 1.00 (ref) | 0.088 |
|  |  | GC | 148/150 | 1.06 (0.76-1.48) |  | 127/126 | 0.94 (0.65-1.36) |  | 104/148 | 1.56 (0.77-3.15) |  | 115/128 | 0.46 (0.22-0.96) |  |
|  |  | CC | 47/52 | 0.95 (0.59-1.52) |  | 44/33 | 1.17 (0.68-2.01) |  | 168/214 | 1.75 (0.87-3.50) |  | 154/181 | 0.45 (0.22-0.92) |  |
|  |  | Per copy variant allele |  | 0.99 (0.80-1.24) | 0.95 |  | 1.04 (0.81-1.33) | 0.76 |  | 1.21 (0.94-1.57) | 0.15 |  | 0.81 (0.61-1.06) | 0.12 |
|  |  | GC/CC vs. GG | 195/202 | 1.03 (0.75-1.41) | 0.86 | 171/159 | 0.99 (0.70-1.39) | 0.96 | 272/362 | 1.66 (0.84-3.28) | 0.15 | 269/309 | 0.45 (0.23-0.92) | **0.03** |
|  |  | CC vs. GG/GC | 284/298 | 0.92 (0.59-1.43) | 0.72 | 262/255 | 1.20 (0.72-2.01) | 0.48 | 117/179 | 1.19 (0.87-1.63) | 0.29 | 143/141 | 0.87 (0.62-1.22) | 0.43 |
| ALOX5AP | rs4769872 | GG | 287/293 | 1.00 (ref) | 0.12 | 263/239 | 1.00 (ref) | 0.55 | 170/242 | 1.00 (ref) | 0.92 | 181/188 | 1.00 (ref) | 0.31 |
|  |  | GA | 42/47 | 0.90 (0.57-1.44) |  | 41/45 | 0.77 (0.47-1.26) |  | 94/121 | 1.07 (0.77-1.50) |  | 109/116 | 0.98 (0.70-1.39) |  |
|  |  | AA | 1/8 | 0.11 (0.01-0.94) |  | 2/2 | 1.33 (0.18-9.99) |  | 21/29 | 1.00 (0.55-1.81) |  | 8/17 | 0.49 (0.20-1.22) |  |
|  |  | Per copy variant allele |  | 0.72 (0.48-1.07) | 0.10 |  | 0.83 (0.53-1.30) | 0.41 |  | 1.03 (0.81-1.31) | 0.82 |  | 0.87 (0.65-1.16) | 0.35 |
|  |  | GA/AA vs. GG | 43/55 | 0.78 (0.50-1.22) | 0.27 | 43/47 | 0.79 (0.49-1.28) | 0.34 | 115/150 | 1.06 (0.77-1.45) | 0.73 | 117/133 | 0.92 (0.66-1.29) | 0.64 |
|  |  | AA vs. GG/GA | 329/340 | 0.12 (0.01-0.95) | **0.045** | 304/284 | 1.39 (0.19-10.41) | 0.75 | 264/363 | 0.97 (0.54-1.75) | 0.92 | 290/304 | 0.50 (0.20-1.21) | 0.13 |
| ALOX5AP | rs9315045 | TT | 181/194 | 1.00 (ref) | 0.94 | 174/162 | 1.00 (ref) | 0.91 | 92/159 | 1.00 (ref) | 0.10 | 92/101 | 1.00 (ref) | 0.71 |
|  |  | TC | 121/130 | 1.02 (0.73-1.42) |  | 109/108 | 0.94 (0.66-1.35) |  | 135/167 | 1.33 (0.94-1.88) |  | 162/169 | 1.08 (0.75-1.56) |  |
|  |  | CC | 28/25 | 1.11 (0.61-2.03) |  | 23/18 | 1.07 (0.54-2.13) |  | 59/63 | 1.57 (1.01-2.45) |  | 43/51 | 0.89 (0.53-1.49) |  |
|  |  | Per copy variant allele |  | 1.04 (0.81-1.33) | 0.76 |  | 0.99 (0.75-1.30) | 0.94 |  | 1.26 (1.02-1.57) | **0.03** |  | 0.97 (0.75-1.24) | 0.79 |
|  |  | TC/CC vs. TT | 149/155 | 1.04 (0.76-1.42) | 0.83 | 132/126 | 0.96 (0.68-1.35) | 0.82 | 194/230 | 1.39 (1.01-1.93) | **0.04** | 205/220 | 1.03 (0.73-1.47) | 0.85 |
|  |  | CC vs. TT/TC | 302/324 | 1.10 (0.61-1.98) | 0.74 | 283/270 | 1.10 (0.56-2.15) | 0.78 | 227/326 | 1.34 (0.90-1.99) | 0.14 | 254/270 | 0.85 (0.54-1.34) | 0.47 |
| ALOX5AP | rs12431114 | AA | 285/301 | 1.00 (ref) | 0.60 | 265/252 | 1.00 (ref) | 0.85 | 259/359 | 1.00 (ref) | 0.89 | 277/294 | 1.00 (ref) | 0.80 |
|  |  | AG | 45/45 | 1.07 (0.68-1.69) |  | 39/33 | 1.13 (0.67-1.91) |  | 25/33 | 1.09 (0.63-1.89) |  | 20/27 | 0.81 (0.44-1.5) |  |
|  |  | GG | 1/4 | 0.34 (0.04-3.07) |  | 2/2 | 0.69 (0.08-5.87) |  | 1/1 | 1.71 (0.11-27.73) |  | 0/1 |  |  |
|  |  | Per copy variant allele |  | 0.96 (0.64-1.46) | 0.86 |  | 1.07 (0.66-1.72) | 0.79 |  | 1.12 (0.67-1.87) | 0.67 |  | 0.76 (0.42-1.37) | 0.36 |
|  |  | AG/GG vs. AA | 46/49 | 1.02 (0.65-1.58) | 0.94 | 41/35 | 1.10 (0.66-1.84) | 0.71 | 26/34 | 1.11 (0.65-1.90) | 0.71 | 20/28 | 0.78 (0.42-1.44) | 0.42 |
|  |  | GG vs. AA/AG | 330/346 | 0.34 (0.04-3.04) | 0.33 | 304/285 | 0.68 (0.08-5.76) | 0.72 | 284/392 | 1.70 (0.11-27.47) | 0.71 |  |  |  |
| ALOX5AP | rs4360791 | GG | 93/104 | 1.00 (ref) | 0.19 | 88/92 | 1.00 (ref) | 0.38 | 51/66 | 1.00 (ref) | 0.85 | 60/69 | 1.00 (ref) | 0.76 |
|  |  | GA | 163/187 | 0.94 (0.65-1.34) |  | 150/126 | 1.31 (0.88-1.95) |  | 136/176 | 0.99 (0.64-1.52) |  | 135/152 | 0.97 (0.63-1.49) |  |
|  |  | AA | 75/56 | 1.38 (0.87-2.19) |  | 67/68 | 1.08 (0.68-1.74) |  | 98/140 | 0.90 (0.58-1.42) |  | 100/98 | 1.12 (0.70-1.77) |  |
|  |  | Per copy variant allele |  | 1.14 (0.91-1.44) | 0.24 |  | 1.06 (0.84-1.34) | 0.64 |  | 0.94 (0.76-1.17) | 0.61 |  | 1.06 (0.85-1.34) | 0.59 |
|  |  | GA/AA vs. GG | 238/243 | 1.04 (0.74-1.46) | 0.83 | 217/194 | 1.23 (0.85-1.79) | 0.27 | 234/316 | 0.95 (0.63-1.43) | 0.81 | 235/250 | 1.03 (0.69-1.54) | 0.90 |
|  |  | AA vs. GG/GA | 256/291 | 1.44 (0.97-2.14) | 0.07 | 238/218 | 0.92 (0.61-1.38) | 0.68 | 187/242 | 0.91 (0.66-1.26) | 0.57 | 195/221 | 1.14 (0.80-1.62) | 0.47 |
| ALOX5AP | rs9579648 | GG | 239/240 | 1.00 (ref) | 0.61 | 214/201 | 1.00 (ref) | 0.95 | 196/250 | 1.00 (ref) | 0.20 | 198/223 | 1.00 (ref) | 0.31 |
|  |  | GC | 83/96 | 0.89 (0.62-1.26) |  | 86/81 | 0.94 (0.64-1.38) |  | 76/128 | 0.77 (0.55-1.09) |  | 92/89 | 1.16 (0.81-1.66) |  |
|  |  | CC | 8/13 | 0.69 (0.28-1.71) |  | 5/6 | 0.90 (0.26-3.17) |  | 13/12 | 1.40 (0.62-3.16) |  | 8/4 | 2.39 (0.69-8.33) |  |
|  |  | Per copy variant allele |  | 0.87 (0.65-1.16) | 0.33 |  | 0.95 (0.68-1.33) | 0.75 |  | 0.91 (0.69-1.21) | 0.53 |  | 1.24 (0.90-1.71) | 0.18 |
|  |  | GC/CC vs. GG | 91/109 | 0.86 (0.62-1.21) | 0.40 | 91/87 | 0.94 (0.65-1.37) | 0.75 | 89/140 | 0.82 (0.59-1.15) | 0.25 | 100/93 | 1.21 (0.85-1.72) | 0.28 |
|  |  | CC vs. GG/GC | 322/336 | 0.71 (0.29-1.76) | 0.47 | 300/282 | 0.92 (0.26-3.21) | 0.89 | 272/378 | 1.52 (0.68-3.41) | 0.31 | 290/312 | 2.29 (0.66-7.94) | 0.19 |
| ALOX5AP | rs9315048 | GG | 185/211 | 1.00 (ref) | 0.09 | 183/177 | 1.00 (ref) | 0.93 | 124/198 | 1.00 (ref) | 0.11 | 146/156 | 1.00 (ref) | 0.90 |
|  |  | GT | 120/125 | 1.12 (0.80-1.55) |  | 100/95 | 1.07 (0.74-1.54) |  | 124/160 | 1.22 (0.88-1.69) |  | 121/134 | 0.93 (0.66-1.32) |  |
|  |  | TT | 26/13 | 2.20 (1.08-4.51) |  | 23/16 | 1.09 (0.54-2.21) |  | 36/33 | 1.72 (1.02-2.91) |  | 27/30 | 0.90 (0.50-1.62) |  |
|  |  | Per copy variant allele |  | 1.27 (0.98-1.65) | 0.07 |  | 1.05 (0.80-1.39) | 0.71 |  | 1.28 (1.01-1.61) | **0.04** |  | 0.94 (0.73-1.21) | 0.65 |
|  |  | GT/TT vs. GG | 146/138 | 1.22 (0.89-1.67) | 0.22 | 123/111 | 1.07 (0.76-1.51) | 0.70 | 160/193 | 1.30 (0.96-1.78) | 0.092 | 148/164 | 0.93 (0.67-1.29) | 0.66 |
|  |  | TT vs. GG/GT | 305/336 | 2.11 (1.05-4.28) | **0.04** | 283/272 | 1.07 (0.53-2.14) | 0.85 | 248/358 | 1.56 (0.95-2.59) | 0.081 | 267/290 | 0.93 (0.53-1.63) | 0.80 |
| ALOX5AP | rs9741777 | AA | 309/334 | 1.00 (ref) | 0.54 | 287/273 | 1.00 (ref) | 0.99 | 276/366 | 1.00 (ref) | 0.19 | 275/299 | 1.00 (ref) | 0.83 |
|  |  | AG | 21/15 | 1.48 (0.73-3.00) |  | 19/14 | 1.06 (0.50-2.25) |  | 10/26 | 0.50 (0.24-1.06) |  | 23/23 | 1.07 (0.57-2.01) |  |
|  |  | GG | 1/1 | 1.44 (0.09-23.17) |  | 0/1 |  |  | 0/1 |  |  | 0/0 |  |  |
|  |  | Per copy variant allele |  | 1.42 (0.75-2.68) | 0.28 |  | 0.92 (0.45-1.85) | 0.81 |  | 0.48 (0.23-0.99) | **0.047** |  | 1.07 (0.57-2.01) | 0.83 |
|  |  | AG/GG vs. AA | 22/16 | 1.48 (0.74-2.94) | 0.27 | 19/15 | 0.98 (0.47-2.06) | 0.96 | 10/27 | 0.48 (0.23-1.01) | 0.054 | 23/23 | 1.07 (0.57-2.01) | 0.83 |
|  |  | GG vs. AA/AG | 330/349 | 1.41 (0.09-22.69) | 0.81 |  |  |  |  |  |  |  |  |  |
| ALOX5AP | rs1132340 | AA | 302/307 | 1.00 (ref) | 0.27 | 276/253 | 1.00 (ref) | 0.26 | 207/291 | 1.00 (ref) | 0.90 | 224/231 | 1.00 (ref) | 0.13 |
|  |  | AG | 26/40 | 0.64 (0.38-1.1) |  | 30/34 | 0.73 (0.42-1.27) |  | 72/92 | 1.07 (0.75-1.53) |  | 69/78 | 0.94 (0.64-1.38) |  |
|  |  | GG | 0/3 |  |  | 0/0 |  |  | 5/8 | 0.87 (0.28-2.73) |  | 4/13 | 0.30 (0.09-0.98) |  |
|  |  | Per copy variant allele |  | 0.58 (0.35-0.97) | **0.04** |  | 0.73 (0.42-1.27) | 0.26 |  | 1.03 (0.75-1.41) | 0.85 |  | 0.79 (0.57-1.09) | 0.15 |
|  |  | AG/GG vs. AA | 26/43 | 0.60 (0.35-1.02) | 0.06 | 30/34 | 0.73 (0.42-1.27) | 0.26 | 77/100 | 1.06 (0.74-1.50) | 0.76 | 73/91 | 0.84 (0.58-1.23) | 0.37 |
|  |  | GG vs. AA/AG |  |  |  |  |  |  | 279/383 | 0.86 (0.27-2.67) | 0.79 | 293/309 | 0.31 (0.09-0.99) | **0.048** |
| ALOX12 | rs3840880^f^ | TT | 103/109 | 1.00 (ref) | 0.67 | 118/78 | 1.00 (ref) | **0.006** | 62/87 | 1.00 (ref) | 0.56 | 71/72 | 1.00 (ref) | 0.35 |
|  |  | TG | 172/173 | 1.11 (0.78-1.58) |  | 141/143 | 0.62 (0.42-0.92) |  | 141/177 | 1.10 (0.74-1.64) |  | 138/168 | 0.86 (0.57-1.30) |  |
|  |  | GG | 54/64 | 0.92 (0.58-1.47) |  | 47/67 | 0.47 (0.28-0.77) |  | 82/129 | 0.90 (0.59-1.39) |  | 89/82 | 1.15 (0.72-1.82) |  |
|  |  | Per copy variant allele |  | 0.98 (0.78-1.23) | 0.87 |  | 0.68 (0.53-0.86) | **0.002** |  | 0.94 (0.76-1.16) | 0.57 |  | 1.08 (0.86-1.36) | 0.51 |
|  |  | TG/GG vs. TT | 226/237 | 1.06 (0.76-1.48) | 0.74 | 188/210 | 0.57 (0.40-0.83) | **0.003** | 223/306 | 1.02 (0.70-1.48) | 0.92 | 227/250 | 0.95 (0.65-1.40) | 0.81 |
|  |  | GG vs. TT/TG | 275/282 | 0.87 (0.58-1.31) | 0.49 | 259/221 | 0.62 (0.40-0.96) | **0.03** | 203/264 | 0.85 (0.60-1.18) | 0.33 | 209/240 | 1.27 (0.88-1.83) | 0.20 |
| ALOX12 | rs2292350 | GG | 109/100 | 1.00 (ref) | 0.56 | 89/107 | 1.00 (ref) | 0.06 | 227/304 | 1.00 (ref) | 0.41 | 235/238 | 1.00 (ref) | 0.24 |
|  |  | GA | 164/187 | 0.83 (0.58-1.18) |  | 149/131 | 1.41 (0.95-2.07) |  | 55/80 | 0.90 (0.61-1.34) |  | 57/79 | 0.71 (0.47-1.06) |  |
|  |  | AA | 57/61 | 0.84 (0.53-1.34) |  | 67/49 | 1.76 (1.08-2.86) |  | 3/9 | 0.42 (0.11-1.59) |  | 5/5 | 1.02 (0.28-3.71) |  |
|  |  | Per copy variant allele |  | 0.90 (0.72-1.13) | 0.38 |  | 1.34 (1.05-1.70) | **0.02** |  | 0.83 (0.58-1.17) | 0.28 |  | 0.77 (0.54-1.11) | 0.16 |
|  |  | GA/AA vs. GG | 221/248 | 0.83 (0.59-1.16) | 0.28 | 216/180 | 1.50 (1.04-2.16) | **0.03** | 58/89 | 0.86 (0.58-1.26) | 0.43 | 62/84 | 0.72 (0.49-1.08) | 0.11 |
|  |  | AA vs. GG/GA | 273/287 | 0.94 (0.62-1.42) | 0.78 | 238/238 | 1.44 (0.93-2.21) | 0.10 | 282/384 | 0.43 (0.11-1.64) | 0.22 | 292/317 | 1.13 (0.31-4.08) | 0.85 |
| ALOX12 | rs1126667^f^ | GG | 111/110 | 1.00 (ref) | 0.76 | 116/81 | 1.00 (ref) | **0.02** | 128/167 | 1.00 (ref) | 0.81 | 136/131 | 1.00 (ref) | 0.11 |
|  |  | GA | 168/175 | 1.02 (0.72-1.45) |  | 142/143 | 0.65 (0.44-0.97) |  | 125/179 | 0.90 (0.65-1.25) |  | 122/149 | 0.71 (0.50-1.01) |  |
|  |  | AA | 51/63 | 0.87 (0.54-1.38) |  | 48/63 | 0.53 (0.32-0.87) |  | 31/46 | 0.90 (0.54-1.51) |  | 40/40 | 1.05 (0.63-1.76) |  |
|  |  | Per copy variant allele |  | 0.95 (0.75-1.19) | 0.63 |  | 0.72 (0.56-0.91) | **0.007** |  | 0.93 (0.74-1.18) | 0.56 |  | 0.93 (0.73-1.18) | 0.53 |
|  |  | GA/AA vs. GG | 219/238 | 0.98 (0.70-1.37) | 0.90 | 190/206 | 0.62 (0.43-0.89) | **0.01** | 156/225 | 0.90 (0.66-1.23) | 0.52 | 162/189 | 0.78 (0.56-1.08) | 0.14 |
|  |  | AA vs. GG/GA | 279/285 | 0.86 (0.57-1.30) | 0.47 | 258/224 | 0.68 (0.44-1.05) | 0.083 | 253/346 | 0.95 (0.58-1.54) | 0.83 | 258/280 | 1.24 (0.77-2.02) | 0.38 |
| ALOX12 | rs434473^f^ | AA | 116/111 | 1.00 (ref) | 0.76 | 114/79 | 1.00 (ref) | 0.018 | 191/260 | 1.00 (ref) | 0.80 | 202/214 | 1.00 (ref) | 0.47 |
|  |  | AG | 164/175 | 0.99 (0.70-1.41) |  | 144/144 | 0.66 (0.44-0.97) |  | 83/109 | 1.03 (0.73-1.46) |  | 80/95 | 0.86 (0.60-1.25) |  |
|  |  | GG | 50/62 | 0.85 (0.53-1.36) |  | 47/63 | 0.50 (0.30-0.83) |  | 12/22 | 0.80 (0.38-1.66) |  | 16/13 | 1.39 (0.64-3.03) |  |
|  |  | Per copy variant allele |  | 0.93 (0.74-1.17) | 0.55 |  | 0.70 (0.55-0.90) | **0.005** |  | 0.96 (0.74-1.26) | 0.79 |  | 1.00 (0.75-1.33) | 0.99 |
|  |  | AG/GG vs. AA | 214/237 | 0.95 (0.68-1.33) | 0.78 | 191/207 | 0.61 (0.42-0.88) | **0.009** | 95/131 | 1.00 (0.72-1.38) | 0.98 | 96/108 | 0.92 (0.65-1.31) | 0.65 |
|  |  | GG vs. AA/AG | 280/286 | 0.85 (0.56-1.30) | 0.46 | 258/223 | 0.65 (0.42-1.01) | 0.056 | 274/369 | 0.79 (0.38-1.63) | 0.52 | 282/309 | 1.45 (0.67-3.15) | 0.34 |
| ALOX12 | rs1042357^f^ | GG | 107/108 | 1.00 (ref) | 0.69 | 112/76 | 1.00 (ref) | 0.011 | 105/128 | 1.00 (ref) | 0.30 | 99/95 | 1.00 (ref) | 0.30 |
|  |  | GT | 170/178 | 1.06 (0.74-1.51) |  | 144/148 | 0.61 (0.41-0.91) |  | 132/179 | 0.89 (0.63-1.26) |  | 144/171 | 0.77 (0.53-1.11) |  |
|  |  | TT | 51/63 | 0.87 (0.55-1.40) |  | 49/64 | 0.50 (0.30-0.82) |  | 47/83 | 0.70 (0.45-1.10) |  | 55/56 | 0.98 (0.61-1.59) |  |
|  |  | Per copy variant allele |  | 0.95 (0.76-1.20) | 0.69 |  | 0.69 (0.54-0.89) | **0.004** |  | 0.85 (0.68-1.05) | 0.13 |  | 0.95 (0.75-1.21) | 0.70 |
|  |  | GT/TT vs. GG | 221/241 | 1.01 (0.72-1.41) | 0.96 | 193/212 | 0.58 (0.40-0.84) | **0.004** | 179/262 | 0.83 (0.60-1.15) | 0.26 | 199/227 | 0.82 (0.58-1.16) | 0.26 |
|  |  | TT vs. GG/GT | 277/286 | 0.84 (0.56-1.28) | 0.43 | 256/224 | 0.67 (0.43-1.04) | 0.073 | 237/307 | 0.75 (0.50-1.12) | 0.16 | 243/266 | 1.15 (0.76-1.76) | 0.50 |
| ALOX12 | rs312462 | CC | 269/270 | 1.00 (ref) | 0.44 | 249/223 | 1.00 (ref) | 0.37 | 251/340 | 1.00 (ref) | 0.95 | 267/287 | 1.00 (ref) | 0.72 |
|  |  | CT | 58/72 | 0.83 (0.56-1.24) |  | 54/63 | 0.75 (0.48-1.15) |  | 34/51 | 0.95 (0.60-1.52) |  | 28/35 | 0.80 (0.46-1.37) |  |
|  |  | TT | 4/8 | 0.54 (0.16-1.86) |  | 3/2 | 1.44 (0.22-9.31) |  | 1/2 | 0.71 (0.06-8.03) |  | 3/0 |  |  |
|  |  | Per copy variant allele |  | 0.80 (0.57-1.13) | 0.21 |  | 0.81 (0.54-1.20) | 0.29 |  | 0.94 (0.61-1.46) | 0.78 |  | 0.96 (0.58-1.58) | 0.88 |
|  |  | CT/TT vs. CC | 62/80 | 0.81 (0.55-1.18) | 0.27 | 57/65 | 0.77 (0.50-1.17) | 0.22 | 35/53 | 0.95 (0.60-1.50) | 0.81 | 31/35 | 0.87 (0.51-1.48) | 0.61 |
|  |  | TT vs. CC/CT | 327/342 | 0.56 (0.16-1.92) | 0.36 | 303/286 | 1.53 (0.24-9.88) | 0.66 | 285/391 | 0.72 (0.06-8.07) | 0.79 |  |  |  |

^a^ OR, odds ratio; 95%CI, 95% confidence interval

^b^ Adjusted for age at diagnosis, family history of breast cancer in a first-degree relative, and proportion of European ancestry

^c^ *P*-trend for genetic dose response determined by coding genotypes as having 0, 1, or 2 variant allele, which was subsequently analyzed as an ordinal variable

^d^ *P* for heterogeneity from dominant or recessive models
^e^ All significant p-values were further adjusted for multiple comparisons using Bonferroni correction, with *P*<0.002 (0.05/30) considered statistically significant.

^f^ Several SNPs on the *ALOX12* gene, rs3840880, rs1126667, rs434473, rs1042357, were found in high LD with rs3840880 (r^2^>0.98) in white women, with a similar association pattern

**Supplemental Table S3. SNPs inflammation-related pathways and risk of breast cancer by ER status in the WCHS^a^**

| **Gene** | **SNP** | **Genotype** | **White** | | | | | | **Black** | | | | | |
| --- | --- | --- | --- | --- | --- | --- | --- | --- | --- | --- | --- | --- | --- | --- |
|  |  |  | **Estrogen Receptor Positive** | | | **Estrogen Receptor Negative** | | | **Estrogen Receptor Positive** | | | **Estrogen Receptor Negative** | | |
|  |  |  | **#Case/**  **Control** | **OR**  **(95% CI)^b.c^** | **P^d,e,f^** | **#Case/**  **Control** | **OR**  **(95% CI)^b,c^** | **P^d,e,f^** | **#Case/**  **Control** | **OR**  **(95% CI)^b,c^** | **P^d,e,f^** | **#Case/**  **Control** | **OR**  **(95% CI)^b,c^** | **P^d,e,f^** |
| COX2 | rs689470 | CC | 410/618 | 1.00 (ref) | **.0006** | 99/618 | 1.00 (ref) | 0.82 | 131/262 | 1.00 (ref) | 0.61 | 51/262 | 1.00 (ref) | 0.46 |
|  |  | CT | 39/17 | 3.05 (1.66-5.59) |  | 5/17 | 1.41 (0.48-4.19) |  | 168/325 | 1.05 (0.78-1.39) |  | 77/325 | 1.18 (0.80-1.75) |  |
|  |  | TT | 1/3 | 0.08 (0.00-3.11) |  | 0/3 |  |  | 52/127 | 0.86 (0.58-1.28) |  | 35/127 | 1.36 (0.83-2.21) |  |
|  |  | Per copy variant allele |  | 2.09 (1.22-3.56) | **0.007** |  | 0.88 (0.31-2.51) | 0.82 |  | 0.95 (0.79-1.15) | 0.60 |  | 1.17 (0.92-1.48) | 0.21 |
|  |  | CT/TT vs. CC | 40/20 | 2.60 (1.46-4.63) | **0.001** | 5/20 | 1.17 (0.39-3.50) | 0.78 | 220/452 | 1.00 (0.76-1.31) | 0.98 | 112/452 | 1.23 (0.85-1.78) | 0.28 |
|  |  | TT vs. CC/CT | 449/635 | 0.07 (0.00-3.08) | 0.17 |  |  |  | 299/587 | 0.84 (0.59-1.20) | 0.34 | 128/587 | 1.23 (0.80-1.89) | 0.34 |
| COX2 | rs2206593 | GG | 390/542 | 1.00 (ref) | 0.22 | 89/542 | 1.00 (ref) | 0.93 | 348/701 | 1.00 (ref) | 0.13 | 162/701 | 1.00 (ref) | 0.88 |
|  |  | GA | 52/89 | 0.79 (0.54-1.15) |  | 14/89 | 1.00 (0.55-1.85) |  | 2/13 | 0.31 (0.07-1.42) |  | 3/13 | 1.10 (0.31-3.99) |  |
|  |  | AA | 5/4 | 2.19 (0.58-8.29) |  | 1/4 | 1.55 (0.17-14.37) |  | 0/0 |  |  | 0/0 |  |  |
|  |  | Per copy variant allele |  | 0.91 (0.65-1.27) | 0.58 |  | 1.05 (0.61-1.82) | 0.86 |  | 0.31 (0.07-1.42) | 0.13 |  | 1.10 (0.31-3.99) | 0.88 |
|  |  | GA/AA vs. GG | 57/93 | 0.84 (0.58-1.21) | 0.35 | 15/93 | 1.03 (0.57-1.86) | 0.93 | 2/13 | 0.31 (0.07-1.42) | 0.13 | 3/13 | 1.10 (0.31-3.99) | 0.88 |
|  |  | AA vs. GG/GA | 442/631 | 2.26 (0.60-8.54) | 0.23 | 103/631 | 1.55 (0.17-14.34) | 0.70 |  |  |  |  |  |  |
| COX2 | rs5275 | TT | 179/279 | 1.00 (ref) | 0.70 | 46/279 | 1.00 (ref) | 0.90 | 62/119 | 1.00 (ref) | 0.76 | 23/119 | 1.00 (ref) | 0.056 |
|  |  | TC | 214/288 | 1.11 (0.85-1.45) |  | 47/288 | 0.96 (0.62-1.50) |  | 175/337 | 0.97 (0.67-1.39) |  | 65/337 | 0.98 (0.58-1.66) |  |
|  |  | CC | 54/70 | 1.14 (0.75-1.72) |  | 11/70 | 0.84 (0.41-1.74) |  | 110/251 | 0.88 (0.60-1.30) |  | 74/251 | 1.52 (0.90-2.56) |  |
|  |  | Per copy variant allele |  | 1.08 (0.90-1.30) | 0.42 |  | 0.93 (0.68-1.28) | 0.67 |  | 0.93 (0.77-1.13) | 0.47 |  | 1.30 (1.01-1.68) | **0.04** |
|  |  | TC/CC vs. TT | 268/358 | 1.11 (0.86-1.44) | 0.40 | 58/358 | 0.94 (0.62-1.43) | 0.76 | 285/588 | 0.93 (0.66-1.31) | 0.68 | 139/588 | 1.20 (0.74-1.96) | 0.46 |
|  |  | CC vs. TT/TC | 393/567 | 1.08 (0.73-1.59) | 0.71 | 93/567 | 0.86 (0.43-1.71) | 0.66 | 237/456 | 0.90 (0.68-1.19) | 0.47 | 88/456 | 1.54 (1.08-2.19) | **0.02** |
| COX2 | rs4648274 | AA | 446/633 | 1.00 (ref) | 0.16 | 103/633 | 1.00 (ref) | 0.70 | 274/541 | 1.00 (ref) | 0.81 | 117/541 | 1.00 (ref) | 0.10 |
|  |  | AC | 2/4 | 0.20 (0.02-1.86) |  | 1/4 | 0.58 (0.04-9.44) |  | 69/160 | 0.90 (0.65-1.24) |  | 41/160 | 1.14 (0.76-1.71) |  |
|  |  | CC | 0/0 |  |  | 0/0 |  |  | 5/12 | 0.95 (0.32-2.81) |  | 7/12 | 2.83 (1.07-7.48) |  |
|  |  | Per copy variant allele |  | 0.20 (0.02-1.86) | 0.16 |  | 0.58 (0.04-9.44) | 0.70 |  | 0.92 (0.69-1.22) | 0.55 |  | 1.32 (0.95-1.83) | 0.10 |
|  |  | AC/CC vs. AA | 2/4 | 0.20 (0.02-1.86) | 0.16 | 1/4 | 0.58 (0.04-9.44) | 0.70 | 74/172 | 0.90 (0.66-1.24) | 0.52 | 48/172 | 1.25 (0.86-1.83) | 0.25 |
|  |  | CC vs. AA/AC |  |  |  |  |  |  | 343/701 | 0.98 (0.33-2.87) | 0.97 | 158/701 | 2.74 (1.04-7.21) | **0.04** |
| COX2 | rs2745557 | GG | 288/397 | 1.00 (ref) | 0.51 | 68/397 | 1.00 (ref) | 0.42 | 249/503 | 1.00 (ref) | 0.93 | 130/503 | 1.00 (ref) | 0.097 |
|  |  | GA | 142/203 | 0.97 (0.74-1.28) |  | 27/203 | 0.82 (0.51-1.33) |  | 91/190 | 0.96 (0.71-1.29) |  | 32/190 | 0.63 (0.41-0.96) |  |
|  |  | AA | 19/37 | 0.70 (0.39-1.27) |  | 9/37 | 1.43 (0.65-3.12) |  | 10/21 | 1.10 (0.50-2.39) |  | 4/21 | 0.77 (0.26-2.29) |  |
|  |  | Per copy variant allele |  | 0.91 (0.73-1.13) | 0.38 |  | 1.02 (0.73-1.44) | 0.89 |  | 0.99 (0.77-1.27) | 0.93 |  | 0.70 (0.49-1.00) | 0.052 |
|  |  | GA/AA vs. GG | 161/240 | 0.93 (0.72-1.21) | 0.59 | 36/240 | 0.92 (0.59-1.43) | 0.71 | 101/211 | 0.97 (0.73-1.29) | 0.84 | 36/211 | 0.64 (0.43-0.96) | **0.03** |
|  |  | AA vs. GG/GA | 430/600 | 0.71 (0.40-1.27) | 0.25 | 95/600 | 1.52 (0.70-3.27) | 0.29 | 340/693 | 1.11 (0.51-2.41) | 0.79 | 162/693 | 0.86 (0.29-2.54) | 0.78 |
| COX2 | rs689466 | AA | 298/451 | 1.00 (ref) | 0.17 | 73/451 | 1.00 (ref) | 0.70 | 278/582 | 1.00 (ref) | 0.84 | 140/582 | 1.00 (ref) | 0.54 |
|  |  | AG | 134/169 | 1.26 (0.95-1.67) |  | 26/169 | 0.99 (0.61-1.61) |  | 67/124 | 1.10 (0.79-1.55) |  | 23/124 | 0.77 (0.47-1.25) |  |
|  |  | GG | 15/16 | 1.52 (0.72-3.19) |  | 4/16 | 1.63 (0.52-5.05) |  | 4/7 | 0.92 (0.26-3.23) |  | 2/7 | 1.27 (0.26-6.32) |  |
|  |  | Per copy variant allele |  | 1.25 (0.99-1.58) | 0.06 |  | 1.09 (0.73-1.62) | 0.67 |  | 1.07 (0.79-1.45) | 0.66 |  | 0.84 (0.54-1.29) | 0.43 |
|  |  | AG/GG vs. AA | 149/185 | 1.28 (0.98-1.68) | 0.07 | 30/185 | 1.04 (0.66-1.66) | 0.85 | 71/131 | 1.09 (0.78-1.52) | 0.60 | 25/131 | 0.80 (0.50-1.27) | 0.34 |
|  |  | GG vs. AA/AG | 432/620 | 1.42 (0.68-2.97) | 0.35 | 99/620 | 1.63 (0.53-5.02) | 0.39 | 345/706 | 0.90 (0.26-3.16) | 0.87 | 163/706 | 1.33 (0.27-6.60) | 0.73 |
| ALOX5 | rs6593482 | GG | 304/454 | 1.00 (ref) | 0.21 | 69/454 | 1.00 (ref) | 0.44 | 247/492 | 1.00 (ref) | 0.61 | 100/492 | 1.00 (ref) | 0.23 |
|  |  | GT | 128/164 | 1.25 (0.94-1.65) |  | 31/164 | 1.34 (0.84-2.13) |  | 94/191 | 0.95 (0.71-1.28) |  | 54/191 | 1.33 (0.92-1.93) |  |
|  |  | TT | 15/15 | 1.49 (0.70-3.20) |  | 3/15 | 1.41 (0.40-5.05) |  | 8/23 | 0.67 (0.29-1.53) |  | 8/23 | 1.57 (0.68-3.65) |  |
|  |  | Per copy variant allele |  | 1.24 (0.98-1.57) | 0.08 |  | 1.28 (0.87-1.90) | 0.21 |  | 0.90 (0.70-1.16) | 0.42 |  | 1.30 (0.96-1.75) | 0.09 |
|  |  | GT/TT vs. GG | 143/179 | 1.27 (0.96-1.66) | 0.09 | 34/179 | 1.34 (0.85-2.11) | 0.20 | 102/214 | 0.92 (0.69-1.22) | 0.56 | 62/214 | 1.36 (0.95-1.94) | 0.10 |
|  |  | TT vs. GG/GT | 432/618 | 1.40 (0.66-2.99) | 0.38 | 100/618 | 1.30 (0.37-4.61) | 0.68 | 341/683 | 0.68 (0.30-1.54) | 0.35 | 154/683 | 1.43 (0.62-3.29) | 0.40 |
| ALOX5 | rs7099684 | TT | 287/385 | 1.00 (ref) | 0.21 | 61/385 | 1.00 (ref) | 0.99 | 278/552 | 1.00 (ref) | 0.65 | 136/552 | 1.00 (ref) | 0.40 |
|  |  | TA | 142/229 | 0.81 (0.62-1.06) |  | 38/229 | 1.01 (0.65-1.57) |  | 67/150 | 0.88 (0.63-1.22) |  | 28/150 | 0.75 (0.48-1.17) |  |
|  |  | AA | 21/24 | 1.20 (0.65-2.25) |  | 4/24 | 1.08 (0.36-3.23) |  | 4/12 | 0.73 (0.23-2.31) |  | 2/12 | 0.66 (0.14-3.04) |  |
|  |  | Per copy variant allele |  | 0.91 (0.73-1.14) | 0.42 |  | 1.02 (0.70-1.47) | 0.92 |  | 0.87 (0.65-1.17) | 0.36 |  | 0.76 (0.51-1.14) | 0.18 |
|  |  | TA/AA vs. TT | 163/253 | 0.84 (0.65-1.09) | 0.20 | 42/253 | 1.01 (0.66-1.56) | 0.95 | 71/162 | 0.87 (0.63-1.19) | 0.38 | 30/162 | 0.74 (0.48-1.15) | 0.18 |
|  |  | AA vs. TT/TA | 429/614 | 1.30 (0.70-2.41) | 0.41 | 99/614 | 1.08 (0.36-3.18) | 0.90 | 345/702 | 0.74 (0.23-2.36) | 0.62 | 164/702 | 0.69 (0.15-3.19) | 0.64 |
| ALOX5 | rs1369214 | GG | 141/190 | 1.00 (ref) | 0.53 | 30/190 | 1.00 (ref) | 0.92 | 70/156 | 1.00 (ref) | **0.01** | 30/156 | 1.00 (ref) | 0.19 |
|  |  | GA | 226/310 | 1.03 (0.77-1.37) |  | 51/310 | 1.06 (0.65-1.73) |  | 157/367 | 0.96 (0.68-1.36) |  | 82/367 | 1.15 (0.73-1.83) |  |
|  |  | AA | 80/134 | 0.85 (0.59-1.22) |  | 23/134 | 1.13 (0.62-2.04) |  | 122/189 | 1.49 (1.03-2.15) |  | 54/189 | 1.53 (0.93-2.51) |  |
|  |  | Per copy variant allele |  | 0.93 (0.78-1.12) | 0.45 |  | 1.06 (0.79-1.43) | 0.69 |  | 1.25 (1.04-1.51) | **0.02** |  | 1.25 (0.97-1.60) | 0.08 |
|  |  | GA/AA vs. GG | 306/444 | 0.98 (0.74-1.28) | 0.86 | 74/444 | 1.08 (0.68-1.71) | 0.74 | 279/556 | 1.14 (0.82-1.57) | 0.44 | 136/556 | 1.28 (0.83-1.98) | 0.27 |
|  |  | AA vs. GG/GA | 367/500 | 0.83 (0.60-1.15) | 0.26 | 81/500 | 1.09 (0.66-1.81) | 0.74 | 227/523 | 1.53 (1.15-2.03) | **0.003** | 112/523 | 1.38 (0.95-1.99) | 0.09 |
| ALOX5 | rs2228065 | GG | 448/636 | 1.00 (ref) | 0.57 | 102/636 | 1.00 (ref) | 0.99 | 295/605 | 1.00 (ref) | 0.93 | 143/605 | 1.00 (ref) | 0.80 |
|  |  | GA | 2/2 | 0.44 (0.03-7.47) |  | 0/2 |  |  | 52/104 | 1.01 (0.70-1.45) |  | 22/104 | 0.85 (0.51-1.39) |  |
|  |  | AA | 0/0 |  |  | 0/0 |  |  | 3/6 | 1.32 (0.32-5.45) |  | 1/6 | 0.83 (0.10-7.05) |  |
|  |  | Per copy variant allele |  | 0.44 (0.03-7.47) | 0.57 |  |  |  |  | 1.03 (0.74-1.44) | 0.85 |  | 0.86 (0.54-1.36) | 0.51 |
|  |  | GA/AA vs. GG | 2/2 | 0.44 (0.03-7.47) | 0.57 |  |  |  | 55/110 | 1.02 (0.71-1.46) | 0.92 | 23/110 | 0.85 (0.52-1.38) | 0.50 |
|  |  | AA vs. GG/GA |  |  |  |  |  |  | 347/709 | 1.31 (0.32-5.44) | 0.71 | 165/709 | 0.85 (0.10-7.21) | 0.88 |
| ALOX5 | rs1487562 | CC | 291/428 | 1.00 (ref) | 0.18 | 72/428 | 1.00 (ref) | 0.31 | 210/400 | 1.00 (ref) | 0.26 | 89/400 | 1.00 (ref) | 0.67 |
|  |  | CT | 132/190 | 1.05 (0.80-1.38) |  | 26/190 | 0.84 (0.52-1.36) |  | 120/258 | 0.88 (0.67-1.16) |  | 65/258 | 1.12 (0.79-1.61) |  |
|  |  | TT | 26/20 | 1.80 (0.96-3.34) |  | 6/20 | 1.82 (0.70-4.70) |  | 20/57 | 0.66 (0.38-1.13) |  | 11/57 | 0.85 (0.43-1.69) |  |
|  |  | Per copy variant allele |  | 1.17 (0.94-1.45) | 0.17 |  | 1.04 (0.71-1.52) | 0.84 |  | 0.84 (0.68-1.04) | 0.11 |  | 1.01 (0.77-1.32) | 0.95 |
|  |  | CT/TT vs. CC | 158/210 | 1.12 (0.86-1.46) | 0.39 | 32/210 | 0.93 (0.59-1.46) | 0.75 | 140/315 | 0.84 (0.64-1.09) | 0.19 | 76/315 | 1.07 (0.76-1.51) | 0.68 |
|  |  | TT vs. CC/CT | 423/618 | 1.77 (0.96-3.28) | 0.07 | 98/618 | 1.92 (0.75-4.91) | 0.18 | 330/658 | 0.69 (0.40-1.18) | 0.18 | 154/658 | 0.81 (0.41-1.59) | 0.54 |
| ALOX5 | rs7099874 | GG | 246/281 | 1.00 (ref) | **0.001** | 40/281 | 1.00 (ref) | 0.44 | 248/525 | 1.00 (ref) | 0.56 | 131/525 | 1.00 (ref) | 0.24 |
|  |  | GC | 160/304 | 0.62 (0.47-0.80) |  | 52/304 | 1.20 (0.77-1.88) |  | 91/165 | 1.16 (0.86-1.57) |  | 28/165 | 0.70 (0.45-1.09) |  |
|  |  | CC | 40/48 | 0.96 (0.60-1.54) |  | 11/48 | 1.59 (0.75-3.35) |  | 9/19 | 0.84 (0.37-1.92) |  | 6/19 | 1.26 (0.49-3.25) |  |
|  |  | Per copy variant allele |  | 0.80 (0.65-0.97) | **0.03** |  | 1.24 (0.89-1.73) | 0.21 |  | 1.07 (0.83-1.38) | 0.61 |  | 0.85 (0.60-1.21) | 0.36 |
|  |  | GC/CC vs. GG | 200/352 | 0.66 (0.51-0.85) | **0.001** | 63/352 | 1.26 (0.82-1.93) | 0.30 | 100/184 | 1.12 (0.84-1.50) | 0.44 | 34/184 | 0.76 (0.50-1.15) | 0.19 |
|  |  | CC vs. GG/GC | 406/585 | 1.20 (0.76-1.89) | 0.44 | 92/585 | 1.43 (0.71-2.9) | 0.32 | 339/690 | 0.81 (0.36-1.84) | 0.61 | 159/690 | 1.36 (0.53-3.49) | 0.53 |
| ALOX5 | rs1051713 | CC | 303/440 | 1.00 (ref) | 0.51 | 76/440 | 1.00 (ref) | 0.23 | 236/486 | 1.00 (ref) | 0.44 | 112/486 | 1.00 (ref) | 0.24 |
|  |  | CT | 127/180 | 1.05 (0.79-1.38) |  | 23/180 | 0.76 (0.46-1.25) |  | 103/193 | 1.08 (0.81-1.45) |  | 50/193 | 1.10 (0.76-1.61) |  |
|  |  | TT | 17/16 | 1.52 (0.74-3.12) |  | 5/16 | 1.89 (0.67-5.32) |  | 11/34 | 0.68 (0.33-1.37) |  | 3/34 | 0.39 (0.12-1.28) |  |
|  |  | Per copy variant allele |  | 1.11 (0.88-1.40) | 0.37 |  | 0.97 (0.65-1.45) | 0.87 |  | 0.97 (0.77-1.22) | 0.78 |  | 0.91 (0.66-1.24) | 0.53 |
|  |  | CT/TT vs. CC | 144/196 | 1.09 (0.83-1.42) | 0.54 | 28/196 | 0.85 (0.53-1.36) | 0.50 | 114/227 | 1.02 (0.77-1.35) | 0.87 | 53/227 | 1.00 (0.69-1.44) | 0.98 |
|  |  | TT vs. CC/CT | 430/620 | 1.50 (0.74-3.07) | 0.26 | 99/620 | 2.03 (0.72-5.69) | 0.18 | 339/679 | 0.66 (0.33-1.33) | 0.25 | 162/679 | 0.37 (0.11-1.24) | 0.11 |
| ALOX5AP | rs4076128 | AA | 224/317 | 1.00 (ref) | 0.95 | 55/317 | 1.00 (ref) | 0.78 | 23/44 | 1.00 (ref) | 0.87 | 9/44 | 1.00 (ref) | 0.98 |
|  |  | AG | 187/262 | 0.97 (0.75-1.27) |  | 38/262 | 0.87 (0.56-1.36) |  | 128/250 | 0.96 (0.54-1.68) |  | 55/250 | 0.99 (0.45-2.17) |  |
|  |  | GG | 37/55 | 0.93 (0.58-1.49) |  | 11/55 | 1.08 (0.52-2.24) |  | 196/417 | 0.90 (0.51-1.57) |  | 102/417 | 1.03 (0.48-2.23) |  |
|  |  | Per copy variant allele |  | 0.97 (0.80-1.18) | 0.75 |  | 0.97 (0.70-1.34) | 0.87 |  | 0.94 (0.76-1.18) | 0.60 |  | 1.03 (0.77-1.38) | 0.85 |
|  |  | AG/GG vs. AA | 224/317 | 0.97 (0.75-1.24) | 0.79 | 49/317 | 0.91 (0.60-1.38) | 0.66 | 324/667 | 0.92 (0.54-1.59) | 0.77 | 157/667 | 1.01 (0.47-2.16) | 0.98 |
|  |  | GG vs. AA/AG | 411/579 | 0.94 (0.60-1.48) | 0.79 | 93/579 | 1.15 (0.57-2.31) | 0.70 | 151/294 | 0.93 (0.71-1.23) | 0.62 | 64/294 | 1.04 (0.73-1.49) | 0.82 |
| ALOX5AP | rs4073259 | AA | 175/247 | 1.00 (ref) | 0.93 | 43/247 | 1.00 (ref) | 0.64 | 17/29 | 1.00 (ref) | 0.80 | 4/29 | 1.00 (ref) | 0.48 |
|  |  | AG | 209/293 | 0.98 (0.75-1.29) |  | 42/293 | 0.85 (0.54-1.35) |  | 110/222 | 0.80 (0.41-1.54) |  | 44/222 | 1.28 (0.42-3.87) |  |
|  |  | GG | 65/97 | 0.93 (0.63-1.36) |  | 19/97 | 1.10 (0.61-2.00) |  | 221/462 | 0.81 (0.42-1.56) |  | 117/462 | 1.57 (0.53-4.64) |  |
|  |  | Per copy variant allele |  | 0.97 (0.81-1.16) | 0.73 |  | 1.01 (0.75-1.36) | 0.96 |  | 0.97 (0.76-1.23) | 0.80 |  | 1.23 (0.88-1.72) | 0.22 |
|  |  | AG/GG vs. AA | 274/390 | 0.97 (0.75-1.25) | 0.81 | 61/390 | 0.91 (0.60-1.40) | 0.68 | 331/684 | 0.81 (0.42-1.53) | 0.51 | 161/684 | 1.45 (0.50-4.27) | 0.50 |
|  |  | GG vs. AA/AG | 384/540 | 0.94 (0.66-1.34) | 0.72 | 85/540 | 1.20 (0.69-2.08) | 0.52 | 127/251 | 1.00 (0.75-1.32) | 0.98 | 48/251 | 1.25 (0.85-1.83) | 0.25 |
| ALOX5AP | rs4293222 | GG | 186/277 | 1.00 (ref) | 0.69 | 45/277 | 1.00 (ref) | 0.66 | 30/44 | 1.00 (ref) | 0.30 | 8/44 | 1.00 (ref) | 0.93 |
|  |  | GC | 205/276 | 1.12 (0.86-1.47) |  | 41/276 | 0.94 (0.59-1.48) |  | 132/276 | 0.66 (0.39-1.12) |  | 63/276 | 1.16 (0.51-2.60) |  |
|  |  | CC | 59/85 | 1.03 (0.69-1.53) |  | 18/85 | 1.24 (0.68-2.29) |  | 187/395 | 0.68 (0.41-1.15) |  | 95/395 | 1.16 (0.52-2.59) |  |
|  |  | Per copy variant allele |  | 1.04 (0.87-1.25) | 0.64 |  | 1.07 (0.80-1.44) | 0.64 |  | 0.92 (0.74-1.14) | 0.42 |  | 1.04 (0.77-1.38) | 0.81 |
|  |  | GC/CC vs. GG | 264/361 | 1.10 (0.86-1.42) | 0.45 | 59/361 | 1.01 (0.66-1.54) | 0.96 | 319/671 | 0.67 (0.41-1.11) | 0.12 | 158/671 | 1.16 (0.53-2.54) | 0.71 |
|  |  | CC vs. GG/GC | 391/553 | 0.97 (0.67-1.40) | 0.87 | 86/553 | 1.28 (0.73-2.26) | 0.39 | 162/320 | 0.97 (0.74-1.27) | 0.83 | 71/320 | 1.02 (0.72-1.45) | 0.91 |
| ALOX5AP | rs4769872 | GG | 395/532 | 1.00 (ref) | **0.03** | 90/532 | 1.00 (ref) | 0.66 | 206/430 | 1.00 (ref) | 0.77 | 103/430 | 1.00 (ref) | 0.78 |
|  |  | GA | 53/92 | 0.69 (0.47-1) |  | 13/92 | 0.78 (0.41-1.48) |  | 126/237 | 1.08 (0.81-1.42) |  | 54/237 | 0.90 (0.62-1.30) |  |
|  |  | AA | 1/10 | 0.14 (0.02-1.12) |  | 1/10 | 0.57 (0.07-4.54) |  | 18/46 | 0.89 (0.50-1.58) |  | 9/46 | 0.82 (0.39-1.73) |  |
|  |  | Per copy variant allele |  | 0.62 (0.44-0.88) | **0.007** |  | 0.77 (0.44-1.34) | 0.36 |  | 1.01 (0.81-1.25) | 0.94 |  | 0.90 (0.68-1.20) | 0.48 |
|  |  | GA/AA vs. GG | 54/102 | 0.63 (0.44-0.92) | **0.02** | 14/102 | 0.76 (0.41-1.41) | 0.38 | 144/283 | 1.05 (0.80-1.37) | 0.74 | 63/283 | 0.89 (0.62-1.26) | 0.50 |
|  |  | AA vs. GG/GA | 448/624 | 0.15 (0.02-1.17) | 0.07 | 103/624 | 0.59 (0.07-4.69) | 0.62 | 332/667 | 0.86 (0.49-1.52) | 0.61 | 157/667 | 0.85 (0.40-1.78) | 0.66 |
| ALOX5AP | rs9315045 | TT | 244/356 | 1.00 (ref) | 0.87 | 62/356 | 1.00 (ref) | 0.52 | 115/260 | 1.00 (ref) | 0.74 | 49/260 | 1.00 (ref) | 0.40 |
|  |  | TC | 172/238 | 1.07 (0.82-1.39) |  | 33/238 | 0.80 (0.50-1.26) |  | 179/336 | 1.12 (0.84-1.50) |  | 84/336 | 1.22 (0.83-1.81) |  |
|  |  | CC | 33/43 | 1.07 (0.65-1.77) |  | 9/43 | 1.17 (0.54-2.54) |  | 57/114 | 1.10 (0.74-1.64) |  | 32/114 | 1.39 (0.84-2.29) |  |
|  |  | Per copy variant allele |  | 1.05 (0.86-1.28) | 0.63 |  | 0.95 (0.68-1.33) | 0.77 |  | 1.06 (0.88-1.28) | 0.54 |  | 1.18 (0.93-1.51) | 0.18 |
|  |  | TC/CC vs. TT | 205/281 | 1.07 (0.83-1.37) | 0.60 | 42/281 | 0.86 (0.56-1.31) | 0.48 | 236/450 | 1.11 (0.84-1.47) | 0.44 | 116/450 | 1.26 (0.87-1.83) | 0.22 |
|  |  | CC vs. TT/TC | 416/594 | 1.04 (0.64-1.70) | 0.86 | 95/594 | 1.28 (0.60-2.72) | 0.53 | 294/596 | 1.03 (0.72-1.47) | 0.87 | 133/596 | 1.23 (0.79-1.90) | 0.36 |
| ALOX5AP | rs12431114 | AA | 389/553 | 1.00 (ref) | 0.35 | 88/553 | 1.00 (ref) | 0.80 | 319/653 | 1.00 (ref) | 0.91 | 156/653 | 1.00 (ref) | 0.68 |
|  |  | AG | 60/78 | 1.15 (0.79-1.66) |  | 15/78 | 1.23 (0.67-2.24) |  | 31/60 | 1.11 (0.70-1.76) |  | 10/60 | 0.73 (0.36-1.47) |  |
|  |  | GG | 1/6 | 0.25 (0.03-2.26) |  | 1/6 | 1.04 (0.12-8.90) |  | 0/2 |  |  | 0/2 |  |  |
|  |  | Per copy variant allele |  | 1.02 (0.73-1.44) | 0.90 |  | 1.17 (0.69-1.98) | 0.55 |  | 1.04 (0.66-1.63) | 0.86 |  | 0.69 (0.35-1.36) | 0.29 |
|  |  | AG/GG vs. AA | 61/84 | 1.09 (0.75-1.56) | 0.66 | 16/84 | 1.22 (0.68-2.18) | 0.51 | 31/62 | 1.08 (0.68-1.70) | 0.75 | 10/62 | 0.70 (0.35-1.41) | 0.32 |
|  |  | GG vs. AA/AG | 449/631 | 0.25 (0.03-2.22) | 0.21 | 103/631 | 1.01 (0.12-8.64) | 1.00 |  |  |  |  |  |  |
| ALOX5AP | rs4360791 | GG | 130/196 | 1.00 (ref) | 0.76 | 29/196 | 1.00 (ref) | 0.71 | 68/135 | 1.00 (ref) | 1.00 | 34/135 | 1.00 (ref) | 0.61 |
|  |  | GA | 222/313 | 1.09 (0.82-1.46) |  | 51/313 | 1.12 (0.68-1.84) |  | 162/328 | 1.00 (0.70-1.42) |  | 71/328 | 0.87 (0.55-1.38) |  |
|  |  | AA | 97/124 | 1.13 (0.79-1.62) |  | 24/124 | 1.28 (0.71-2.31) |  | 117/238 | 1.00 (0.69-1.45) |  | 61/238 | 1.05 (0.65-1.69) |  |
|  |  | Per copy variant allele |  | 1.07 (0.89-1.27) | 0.47 |  | 1.13 (0.84-1.52) | 0.41 |  | 1.00 (0.83-1.20) | 0.99 |  | 1.05 (0.83-1.33) | 0.70 |
|  |  | GA/AA vs. GG | 319/437 | 1.10 (0.84-1.45) | 0.47 | 75/437 | 1.17 (0.73-1.86) | 0.51 | 279/566 | 1.00 (0.72-1.39) | 0.98 | 132/566 | 0.95 (0.62-1.44) | 0.79 |
|  |  | AA vs. GG/GA | 352/509 | 1.07 (0.79-1.46) | 0.66 | 80/509 | 1.19 (0.72-1.97) | 0.49 | 230/463 | 1.00 (0.76-1.32) | 1.00 | 105/463 | 1.16 (0.81-1.65) | 0.42 |
| ALOX5AP | rs9579648 | GG | 319/441 | 1.00 (ref) | 0.65 | 69/441 | 1.00 (ref) | 0.82 | 235/473 | 1.00 (ref) | 0.06 | 114/473 | 1.00 (ref) | 0.74 |
|  |  | GC | 121/177 | 0.94 (0.71-1.25) |  | 31/177 | 1.08 (0.68-1.72) |  | 99/217 | 0.97 (0.72-1.29) |  | 47/217 | 0.92 (0.63-1.34) |  |
|  |  | CC | 8/19 | 0.69 (0.29-1.62) |  | 4/19 | 1.38 (0.45-4.20) |  | 16/16 | 2.33 (1.13-4.82) |  | 5/16 | 1.36 (0.49-3.82) |  |
|  |  | Per copy variant allele |  | 0.91 (0.71-1.16) | 0.43 |  | 1.12 (0.76-1.64) | 0.56 |  | 1.14 (0.90-1.46) | 0.28 |  | 0.99 (0.71-1.37) | 0.95 |
|  |  | GC/CC vs. GG | 129/196 | 0.92 (0.70-1.21) | 0.54 | 35/196 | 1.11 (0.71-1.73) | 0.64 | 115/233 | 1.06 (0.80-1.40) | 0.70 | 52/233 | 0.95 (0.66-1.37) | 0.79 |
|  |  | CC vs. GG/GC | 440/618 | 0.70 (0.30-1.64) | 0.41 | 100/618 | 1.35 (0.45-4.06) | 0.60 | 334/690 | 2.36 (1.15-4.84) | **0.02** | 161/690 | 1.40 (0.50-3.90) | 0.52 |
| ALOX5AP | rs9315048 | GG | 256/388 | 1.00 (ref) | 0.26 | 62/388 | 1.00 (ref) | **0.03** | 173/354 | 1.00 (ref) | 0.76 | 72/354 | 1.00 (ref) | 0.37 |
|  |  | GT | 164/220 | 1.17 (0.9-1.52) |  | 30/220 | 0.90 (0.56-1.45) |  | 141/294 | 0.96 (0.73-1.26) |  | 70/294 | 1.15 (0.79-1.65) |  |
|  |  | TT | 30/29 | 1.47 (0.84-2.55) |  | 12/29 | 2.47 (1.19-5.15) |  | 36/63 | 1.14 (0.73-1.80) |  | 20/63 | 1.49 (0.85-2.63) |  |
|  |  | Per copy variant allele |  | 1.19 (0.96-1.46) | 0.11 |  | 1.26 (0.91-1.76) | 0.17 |  | 1.03 (0.84-1.25) | 0.79 |  | 1.20 (0.93-1.55) | 0.17 |
|  |  | GT/TT vs. GG | 194/249 | 1.20 (0.93-1.55) | 0.15 | 42/249 | 1.10 (0.72-1.69) | 0.65 | 177/357 | 0.99 (0.76-1.29) | 0.95 | 90/357 | 1.21 (0.85-1.71) | 0.28 |
|  |  | TT vs. GG/GT | 420/608 | 1.38 (0.80-2.38) | 0.24 | 92/608 | 2.56 (1.25-5.25) | **0.01** | 314/648 | 1.17 (0.75-1.81) | 0.49 | 142/648 | 1.40 (0.82-2.39) | 0.22 |
| ALOX5AP | rs9741777 | AA | 424/607 | 1.00 (ref) | 0.89 | 98/607 | 1.00 (ref) | 0.59 | 332/665 | 1.00 (ref) | 0.55 | 155/665 | 1.00 (ref) | 0.99 |
|  |  | AG | 26/29 | 1.15 (0.65-2.03) |  | 5/29 | 1.10 (0.41-2.91) |  | 19/49 | 0.73 (0.42-1.28) |  | 11/49 | 0.94 (0.48-1.87) |  |
|  |  | GG | 0/2 |  |  | 1/2 | 3.50 (0.31-39.45) |  | 0/1 |  |  | 0/1 |  |  |
|  |  | Per copy variant allele |  | 1.01 (0.59-1.72) | 0.98 |  | 1.32 (0.60-2.88) | 0.49 |  | 0.72 (0.41-1.24) | 0.23 |  | 0.91 (0.47-1.78) | 0.79 |
|  |  | AG/GG vs. AA | 26/31 | 1.08 (0.61-1.89) | 0.80 | 6/31 | 1.24 (0.50-3.06) | 0.64 | 19/50 | 0.72 (0.41-1.26) | 0.25 | 11/50 | 0.93 (0.47-1.83) | 0.83 |
|  |  | GG vs. AA/AG |  |  |  | 103/636 | 3.49 (0.31-39.25) | 0.31 |  |  |  |  |  |  |
| ALOX5AP | rs1132340 | AA | 412/560 | 1.00 (ref) | 0.076 | 96/560 | 1.00 (ref) | 0.51 | 262/522 | 1.00 (ref) | 0.48 | 122/522 | 1.00 (ref) | 0.60 |
|  |  | AG | 35/74 | 0.60 (0.39-0.93) |  | 8/74 | 0.64 (0.30-1.36) |  | 81/170 | 0.95 (0.70-1.30) |  | 40/170 | 0.99 (0.66-1.48) |  |
|  |  | GG | 0/3 |  |  | 0/3 |  |  | 6/21 | 0.57 (0.22-1.44) |  | 3/21 | 0.53 (0.15-1.81) |  |
|  |  | Per copy variant allele |  | 0.58 (0.38-0.88) | **0.01** |  | 0.61 (0.29-1.28) | 0.19 |  | 0.89 (0.68-1.15) | 0.37 |  | 0.90 (0.64-1.26) | 0.53 |
|  |  | AG/GG vs. AA | 35/77 | 0.58 (0.38-0.90) | **0.02** | 8/77 | 0.61 (0.29-1.32) | 0.21 | 87/191 | 0.91 (0.68-1.23) | 0.54 | 43/191 | 0.94 (0.63-1.38) | 0.74 |
|  |  | GG vs. AA/AG |  |  |  |  |  |  | 343/692 | 0.58 (0.23-1.46) | 0.24 | 162/692 | 0.53 (0.15-1.81) | 0.31 |
| ALOX12 | rs3840880^g^ | TT | 154/187 | 1.00 (ref) | 0.27 | 43/187 | 1.00 (ref) | **0.01** | 79/159 | 1.00 (ref) | 0.20 | 39/159 | 1.00 (ref) | 0.07 |
|  |  | TG | 217/316 | 0.87 (0.65-1.15) |  | 51/316 | 0.75 (0.47-1.17) |  | 154/345 | 0.90 (0.64-1.26) |  | 93/345 | 1.05 (0.69-1.60) |  |
|  |  | GG | 78/131 | 0.74 (0.52-1.07) |  | 10/131 | 0.34 (0.16-0.70) |  | 117/211 | 1.19 (0.83-1.70) |  | 34/211 | 0.63 (0.38-1.06) |  |
|  |  | Per copy variant allele |  | 0.86 (0.72-1.03) | 0.10 |  | 0.63 (0.46-0.86) | **0.004** |  | 1.11 (0.92-1.33) | 0.28 |  | 0.80 (0.63-1.02) | 0.07 |
|  |  | TG/GG vs. TT | 295/447 | 0.83 (0.63-1.09) | 0.17 | 61/447 | 0.62 (0.40-0.96) | **0.03** | 271/556 | 1.00 (0.73-1.37) | 0.99 | 127/556 | 0.89 (0.60-1.34) | 0.59 |
|  |  | GG vs. TT/TG | 371/503 | 0.81 (0.59-1.11) | 0.19 | 94/503 | 0.40 (0.20-0.80) | **0.009** | 233/504 | 1.28 (0.96-1.69) | 0.09 | 132/504 | 0.61 (0.41-0.93) | 0.02 |
| ALOX12 | rs2292350 | GG | 142/207 | 1.00 (ref) | 0.77 | 27/207 | 1.00 (ref) | 0.33 | 278/542 | 1.00 (ref) | 0.30 | 127/542 | 1.00 (ref) | 0.91 |
|  |  | GA | 218/318 | 1.03 (0.77-1.36) |  | 55/318 | 1.37 (0.83-2.25) |  | 67/159 | 0.77 (0.55-1.08) |  | 35/159 | 0.99 (0.65-1.52) |  |
|  |  | AA | 88/110 | 1.14 (0.79-1.64) |  | 22/110 | 1.53 (0.83-2.83) |  | 6/14 | 0.79 (0.29-2.12) |  | 2/14 | 0.71 (0.16-3.22) |  |
|  |  | Per copy variant allele |  | 1.06 (0.89-1.27) | 0.51 |  | 1.25 (0.92-1.68) | 0.15 |  | 0.80 (0.60-1.07) | 0.14 |  | 0.95 (0.65-1.39) | 0.81 |
|  |  | GA/AA vs. GG | 306/428 | 1.06 (0.81-1.38) | 0.69 | 77/428 | 1.41 (0.88-2.26) | 0.15 | 73/173 | 0.77 (0.56-1.07) | 0.12 | 37/173 | 0.97 (0.64-1.48) | 0.90 |
|  |  | AA vs. GG/GA | 360/525 | 1.12 (0.81-1.55) | 0.48 | 82/525 | 1.26 (0.75-2.11) | 0.38 | 345/701 | 0.86 (0.32-2.28) | 0.75 | 162/701 | 0.71 (0.16-3.21) | 0.66 |
| ALOX12 | rs1126667^g^ | GG | 154/191 | 1.00 (ref) | 0.43 | 45/191 | 1.00 (ref) | **0.009** | 157/298 | 1.00 (ref) | 0.19 | 82/298 | 1.00 (ref) | 0.14 |
|  |  | GA | 218/318 | 0.88 (0.66-1.17) |  | 50/318 | 0.71 (0.45-1.12) |  | 145/328 | 0.80 (0.61-1.06) |  | 71/328 | 0.79 (0.55-1.13) |  |
|  |  | AA | 77/126 | 0.79 (0.55-1.14) |  | 9/126 | 0.31 (0.15-0.66) |  | 48/86 | 1.09 (0.72-1.64) |  | 13/86 | 0.56 (0.29-1.05) |  |
|  |  | Per copy variant allele |  | 0.89 (0.74-1.06) | 0.20 |  | 0.61 (0.44-0.83) | **0.002** |  | 0.97 (0.80-1.17) | 0.75 |  | 0.76 (0.59-1.00) | **0.046** |
|  |  | GA/AA vs. GG | 295/444 | 0.85 (0.65-1.12) | 0.25 | 59/444 | 0.59 (0.38-0.91) | **0.02** | 193/414 | 0.86 (0.66-1.12) | 0.27 | 84/414 | 0.74 (0.53-1.04) | 0.08 |
|  |  | AA vs. GG/GA | 372/509 | 0.86 (0.62-1.18) | 0.35 | 95/509 | 0.38 (0.19-0.78) | **0.008** | 302/626 | 1.22 (0.83-1.79) | 0.32 | 153/626 | 0.62 (0.34-1.15) | 0.13 |
| ALOX12 | rs434473^g^ | AA | 157/190 | 1.00 (ref) | 0.32 | 46/190 | 1.00 (ref) | **0.006** | 233/474 | 1.00 (ref) | 0.71 | 118/474 | 1.00 (ref) | 0.54 |
|  |  | AG | 216/319 | 0.86 (0.65-1.14) |  | 49/319 | 0.67 (0.43-1.06) |  | 98/204 | 0.95 (0.71-1.27) |  | 43/204 | 0.86 (0.58-1.27) |  |
|  |  | GG | 75/125 | 0.76 (0.53-1.10) |  | 9/125 | 0.30 (0.14-0.65) |  | 20/35 | 1.23 (0.68-2.20) |  | 5/35 | 0.64 (0.24-1.69) |  |
|  |  | Per copy variant allele |  | 0.87 (0.73-1.04) | 0.13 |  | 0.59 (0.43-0.81) | **0.001** |  | 1.02 (0.82-1.28) | 0.84 |  | 0.84 (0.61-1.15) | 0.27 |
|  |  | AG/GG vs. AA | 291/444 | 0.83 (0.64-1.09) | 0.17 | 58/444 | 0.57 (0.37-0.87) | **0.01** | 118/239 | 0.99 (0.75-1.30) | 0.92 | 48/239 | 0.83 (0.57-1.21) | 0.33 |
|  |  | GG vs. AA/AG | 373/509 | 0.84 (0.61-1.16) | 0.29 | 95/509 | 0.38 (0.19-0.78) | **0.008** | 331/678 | 1.25 (0.70-2.22) | 0.45 | 161/678 | 0.67 (0.26-1.75) | 0.41 |
| ALOX12 | rs1042357^g^ | GG | 151/184 | 1.00 (ref) | 0.29 | 43/184 | 1.00 (ref) | **0.02** | 121/223 | 1.00 (ref) | 0.42 | 60/223 | 1.00 (ref) | 0.13 |
|  |  | GT | 219/326 | 0.85 (0.64-1.13) |  | 51/326 | 0.71 (0.45-1.12) |  | 165/350 | 0.82 (0.61-1.10) |  | 82/350 | 0.84 (0.58-1.23) |  |
|  |  | TT | 77/127 | 0.76 (0.52-1.09) |  | 10/127 | 0.35 (0.17-0.72) |  | 65/139 | 0.88 (0.61-1.28) |  | 22/139 | 0.58 (0.34-0.99) |  |
|  |  | Per copy variant allele |  | 0.87 (0.72-1.04) | 0.12 |  | 0.62 (0.45-0.86) | **0.004** |  | 0.92 (0.77-1.11) | 0.39 |  | 0.78 (0.61-1.00) | **0.049** |
|  |  | GT/TT vs. GG | 296/453 | 0.82 (0.63-1.07) | 0.15 | 61/453 | 0.60 (0.39-0.93) | **0.02** | 230/489 | 0.84 (0.64-1.10) | 0.21 | 104/489 | 0.77 (0.54-1.10) | 0.14 |
|  |  | TT vs. GG/GT | 370/510 | 0.84 (0.61-1.16) | 0.28 | 94/510 | 0.42 (0.21-0.84) | **0.01** | 286/573 | 0.99 (0.71-1.39) | 0.97 | 142/573 | 0.64 (0.39-1.04) | 0.074 |
| ALOX12 | rs312462 | CC | 366/493 | 1.00 (ref) | 0.36 | 84/493 | 1.00 (ref) | 0.79 | 311/627 | 1.00 (ref) | 0.29 | 147/627 | 1.00 (ref) | 1.00 |
|  |  | CT | 78/135 | 0.79 (0.58-1.09) |  | 19/135 | 0.85 (0.50-1.45) |  | 36/86 | 0.90 (0.59-1.36) |  | 19/86 | 1.00 (0.59-1.71) |  |
|  |  | TT | 6/10 | 0.98 (0.35-2.78) |  | 1/10 | 0.67 (0.08-5.35) |  | 4/2 | 3.77 (0.65-21.91) |  | 0/2 |  |  |
|  |  | Per copy variant allele |  | 0.84 (0.63-1.11) | 0.22 |  | 0.84 (0.52-1.37) | 0.49 |  | 1.03 (0.71-1.51) | 0.86 |  | 0.96 (0.57-1.62) | 0.89 |
|  |  | CT/TT vs. CC | 84/145 | 0.81 (0.59-1.10) | 0.17 | 20/145 | 0.84 (0.50-1.42) | 0.51 | 40/88 | 0.96 (0.64-1.45) | 0.86 | 19/88 | 0.98 (0.58-1.67) | 0.95 |
|  |  | TT vs. CC/CT | 444/628 | 1.02 (0.36-2.90) | 0.96 | 103/628 | 0.69 (0.09-5.51) | 0.73 | 347/713 | 3.83 (0.66-22.21) | 0.13 |  |  |  |

^a^ Based on 554 (87.0%) white cases and 517 (88.5%) Black cases with available data on ER status

^b^ OR, odds ratio; 95%CI, 95% confidence interval

^c^ Adjusted for age at diagnosis, family history of breast cancer in a first-degree relative, and proportion of European ancestry

^d^ *P*-trend for genetic dose response determined by coding genotypes as having 0, 1, or 2 variant allele, which was subsequently analyzed as an ordinal variable

^e^ *P* for heterogeneity from dominant or recessive models

^f^ All significant p-values were further adjusted for multiple comparisons using Bonferroni correction, with *P*<0.002 (0.05/30) considered statistically significant.

^g^ Several SNPs on the ALOX12 gene, rs3840880, rs1126667, rs434473, rs1042357, were found in high LD with rs3840880 (r^2^>0.98) in white women, with a similar association pattern
